# Development of Magnetic Sponges Using Steel Melting on 3D Carbonized Spongin Scaffolds Under Extreme Biomimetics Conditions

**DOI:** 10.3390/biomimetics10060350

**Published:** 2025-05-28

**Authors:** Bartosz Leśniewski, Martin Kopani, Anna Szczurek, Michał Matczak, Janusz Dubowik, Martyna Kotula, Anita Kubiak, Dmitry Tsurkan, Eliza Romańczuk-Ruszuk, Marek Nowicki, Krzysztof Nowacki, Iaroslav Petrenko, Hermann Ehrlich

**Affiliations:** 1 Center for Advanced Technologies, Adam Mickiewicz University, Uniwersytetu Poznańskiego 10, 61-614 Poznań, Poland; anna.szczurek@amu.edu.pl (A.S.); markot6@amu.edu.pl (M.K.); anikub@amu.edu.pl (A.K.); marek.nowicki@amu.edu.pl (M.N.); 2 Faculty of Chemistry, Adam Mickiewicz University, Uniwersytetu Poznańskiego 8, 61-614 Poznań, Poland; 3 Institute of Medical Physics and Biophysics, Faculty of Medicine, Comenius University, Sasinkova 2, 81272 Bratislava, Slovakia; martin.kopani@fmed.uniba.sk; 4 Institute of Molecular Physics, Polish Academy of Sciences, Mariana Smoluchowskiego 17, 60-179 Poznań, Poland; michmat88@ifmpan.poznan.pl (M.M.); janusz.dubowik@ifmpan.poznan.pl (J.D.); 5 Institute of Nanoscale and Biobased Materials, Faculty of Materials Science and Material Technology, Technische Universität Bergakademie Freiberg, 09599 Freiberg, Germany; tsurkandd@gmail.com; 6 Institute of Biomedical Engineering, Faculty of Mechanical Engineering, Bialystok University of Technology, Wiejska Str. 45C, 15-351 Białystok, Poland; e.romanczuk@pb.edu.pl; 7 Institute of Physics, Faculty of Materials Engineering and Technical Physics, Poznan University of Technology, Piotrowo 3, 60-965 Poznań, Poland; 8 Institute of Chemistry and Technical Electrochemistry, Faculty of Chemical Technology, Poznan University of Technology, Berdychowo 4, 60-965 Poznań, Poland; krzysztof.nowacki@put.poznan.pl; 9 Vice-Rectorate for Research, International Affairs and Transfer, Freiberg University of Mining and Technology, Akademiestrasse 6, 09599 Freiberg, Germany; iaroslav.petrenko@zuv.tu-freiberg.de; 10 Institute of Chemical Technology and Engineering, Faculty of Chemical Technology, Poznan University of Technology, Berdychowo 4, 60-965 Poznań, Poland

**Keywords:** extreme biomimetics, bioinspired materials, spongin scaffold, carbonization, steel melting, composite materials, HER, water splitting

## Abstract

This study presents a novel approach to fabricating magnetic sponge-like composites by melting various types of steel onto three-dimensional (3D) carbonized spongin scaffolds under extreme biomimetic conditions. Spongin, a renewable marine biopolymer with high thermal stability, was carbonized at 1200 °C to form a turbostratic graphite matrix capable of withstanding the high-temperature steel melting process (1450–1600 °C). The interaction between molten steel vapors and the carbonized scaffolds resulted in the formation of nanostructured iron oxide (primarily hematite) coatings, which impart magnetic properties to the resulting composites. Detailed characterization using SEM-EDX, HRTEM, FT-IR, and XRD confirmed the homogeneous distribution of iron oxides on and within the carbonized fibrous matrix. Electrochemical measurements further demonstrated the electrocatalytic potential of the composite, particularly the sample modified with stainless steel 316L—for the hydrogen evolution reaction (HER), offering promising perspectives for green hydrogen production. This work highlights the potential of extreme biomimetics to create functional, scalable, and sustainable materials for applications in catalysis, environmental remediation, and energy technologies.

## 1. Introduction

Biomimetics is an interdisciplinary science that draws inspiration from natural phenomena and biological processes. Biomimetics of materials focuses on the functional properties of naturally occurring materials to develop novel composite materials with specific physicochemical properties, structures, and applications [1,2,3].

Extreme biomimetics is an emerging sub-discipline within materials science, introduced in 2010 by Prof. Hermann Ehrlich. The main principle is the use of renewable biopolymers resistant to extreme environmental conditions of temperature, pressure, and pH. Combining extreme biomimetics with bioinspired materials science makes it possible to obtain innovative composite materials with unique properties suitable for application in the harsh conditions of modern industry. The philosophy of extreme biomimetics is based on four established approaches:

⮚ finding relevant renewable natural sources and examples of inspiration in nature;
⮚ understanding biological principles and mechanisms underlying natural phenomena;
⮚ applying of accessible procedures related to the use of biological materials;
⮚ developing advanced scientific strategies based on available knowledge in order to create a new generation of composite materials [4,5,6].

**Figure 1:**
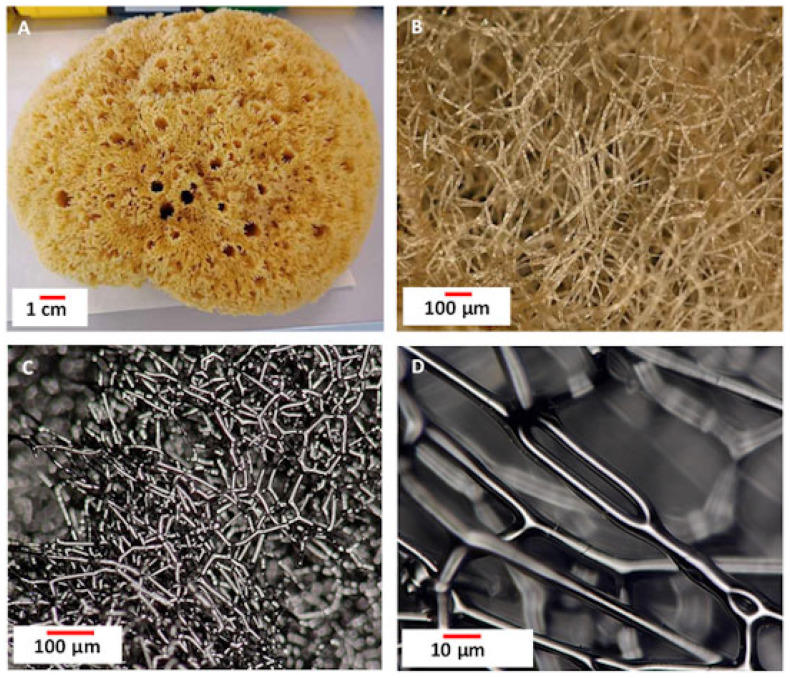
(**A**) The spongin-based skeleton of a *H. communis* marine sponge. Digital microscopy imagery: (**B**) dried skeleton treated with 3 M HCl; (**C**,**D**) skeleton after carbonization at 1200 °C for 1 h in an oxygen-free environment.

This approach has become a milestone in synthesizing innovative composite materials because it enables the creation of functional constructs on the basis of already existing, naturally occurring three-dimensional (3D) micro- and macro-level architectures, such as sponge skeletons. Typical sources include cultivated marine bath sponges (i.e., *Hippospongia communis*) [4,7,8] whose 3D microporous architecture of their skeletons is formed by halogenated collagen-based [9] structural biopolymer spongin (Figure 1). Spongin is already used in extreme biomimetics due to its high thermal stability—up to 360 °C and remarkable chemical resistance [10,11,12,13]. One of spongin’s most intriguing structural properties is its ability to preserve its 3D microarchitecture even after carbonization at temperatures up to 1200 °C [10,11,12,13,14]. Under such extreme conditions, spongin can be transformed into turbostratic graphite with nanopores, which significantly increases the specific surface area of the resulting carbon and enables its efficient applications in catalysis [15,16,17,18]. These extraordinary properties of the spongin-based sponge’s skeletons were the appealing reason for choosing them as a composite scaffold material.

Recently, it was shown that a MnO_2_-based composite, created using a carbonized spongin scaffold, can be applied in the storage and processing of renewable energy [19]. Another spongin-based composite, incorporating Fe_3_O_4_ nanocores, can be used for wastewater treatment and drug delivery [20].

Spongin-based skeletons of marine sponges of the subclass Keratosa (class Demospongiae) play a remarkable role in so-called forced biomineralization processes [21], in which iron oxides, such as lepidocrocite (γ-FeO(OH)), were deposited on their skeletal structures [14,22,23]. This phenomenon results from the biocorrosion of artificial iron-containing materials submerged in seawater near the bath sponges’ habitats. Lepidocrocite, a crystalline iron oxide-hydroxide mineral, is distinguished by its reddish-brown color, sub-metallic luster, and magnetic properties, making it a notable product of this biological interaction [24]. Consequently, those marine bath sponges, whose skeletons are naturally enriched with iron oxides-containing biominerals [3,25,26], represent the source for bioinspired approaches in the development of artificial magnetic sponges.

Man-made magnetic sponges are versatile materials with a wide range of possible applications, including environmental remediation, biomedical engineering, and catalysis [27,28,29]. These materials are typically created by integrating magnetic nanoparticles into porous matrices, thereby imparting properties like hydrophobicity, selective absorption, and catalytic activity. Their tunability in structure and functionality allows for broad adaptability across diverse scientific and industrial disciplines.

For example, magnetic carbon sponges (MCS), derived from carbonized metal-organic frameworks (MOFs) and melamine sponges, exhibit both macroporosity and nanoporosity. The combination of structural porosity and catalytic sites makes these materials particularly valuable for environmental cleanup and energy-related applications [30,31]. In biomedicine, keratin sponges with magnetic MgFe hydrotalcite nanoparticles (HTIc) have been shown to enhance osteoblast activity under static magnetic fields, indicating potential for tissue regeneration [27]. Polyurethane-based magnetic sponges, modified with Fe_3_O_4_ nanoparticles and hydrophobic agents, can selectively absorb oils and organic solvents while repelling water, making them highly effective for marine oil spill cleanup and wastewater treatment [29,31,32]. Furthermore, silicone-based superhydrophobic magnetic sponges, synthesized with Fe_3_O_4_ nanoparticles, show excellent stability and high absorption capacities ranging from 700–1700%, depending on the type of organic substance. Their resistance to harsh chemical environments makes them durable for industrial applications, including chemical spill mitigation [33].

Our previous studies [3,25,34] have demonstrated the versatility of spongin as a scaffold for the development of advanced iron-oxide-based composite materials. One example is the “iron–spongin” composite, developed through a biomimetic approach to create a novel 3D spongin scaffold containing lepidocrocite (γ-FeO(OH)). This process, conducted for the first time under laboratory conditions at a temperature of 24 °C, using iron and artificial seawater, highlights the adaptability of spongin for innovative material design. The resulting magnetic material demonstrated excellent dopamine (DA) sensing performance, further showcasing its potential for advanced applications [25]. In another study, spongin from *H. communis* demosponge served as a template to produce a microporous goethite (α-FeO(OH)) composite using an extreme biomimetic method based on the application of iron powder and crystalline iodine [3]. This material has shown potential in biomedicine and electrochemical dopamine detection [34]. Expanding these efforts, for the first time, 3D spongin scaffolds derived from cultivated *H. communis* were employed for the electro-assisted deposition of iron oxides, such as goethite (α-FeO(OH)) and lepidocrocite (γ-FeO(OH)), enabling a significantly faster process for obtaining composites and advancing their potential applications [26].

In this work, for the first time, we present an extreme biomimetics process of obtaining magnetic sponges by melting selected types of steel on already carbonized at 1200 °C spongin matrices (Figure 1C). During this process, a reaction occurs between the vaporized steel and the carbonized spongin microfibers, leading to the formation of a novel microporous 3D composite material with magnetic properties. We also hypothesize that the proposed method could significantly advance the development of efficient and cost-effective catalysts for electrochemical water splitting. In particular, the unique 3D structure of the sponge-derived precursors, coated with iron oxides, may synergistically enhance the performance of electrodes used in the hydrogen evolution reaction (HER), thereby improving the overall efficiency of the water-splitting process.

## 2. Materials and Methods

### 2.1. Materials

Purified spongin scaffolds isolated from the marine demosponge *Hippospongia communis* (Lamarck, 1814) were obtained from INTIB GmbH (Freiberg, Germany). Four different types of steel shavings or powders have been used. Construction steel EN S235JRG2 (AISI 1015), carbon steel C45, and stainless steel 316L powders were supplied from Białystok University of Technology (Białystok, Poland). No. 172/1 low alloy cast iron was provided by British Chemical Standards (Middlesbrough, United Kingdom) (Table 1). The hydrochloric acid (35–38%) was purchased from Stanlab (Lublin, Poland), and the hydrofluoric acid (40%) was purchased from Chempur (Piekary Śląskie, Poland). Sodium sulfate (>99%); polyvinylidene fluoride (>99%), and N-methyl-2-pyrrolidone (>99%) were purchased from Sigma-Aldrich (Burlington, VT, USA). Redistilled water was used to prepare all aqueous solutions in electrochemical studies.

### 2.2. Sample Preparation

**Figure 2:**
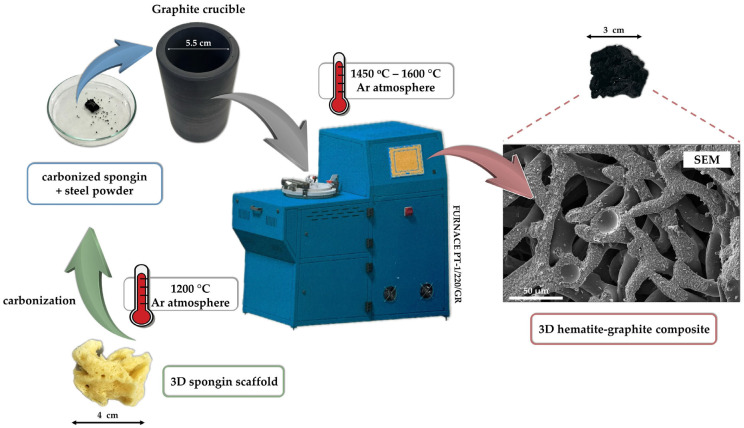
Schematic representation of the methodological approach to creating composites in the form of magnetic sponges.

The purified spongin scaffold was cut into pieces of different sizes and weights and then placed in 3 M hydrochloric acid (Stanlab) for 72 h at room temperature. Next, the spongin pieces were rinsed with distilled water until pH 6.5 was achieved. Then, they were dried in an oven at 40 °C and carbonized in a graphite crucible using a high-temperature furnace Czylok PT-1/220/GR (Poland) for 1 h at 1200 °C under oxygen-free conditions. Selected steels (Table 1) were then melted on the carbonized spongin skeletons in graphite crucibles (Figure 2):

(a) construction steel EN S235JRG2 (AISI 1015), carbon steel C45, stainless steel 316 L powder, and No. 172/1 low alloy cast iron (see Supplementary Materials, Figure S1) were melted on carbonized spongin scaffolds, which were obtained from samples after HCl treatment using the high-temperature furnace during 90 min at 1450 °C under oxygen-free conditions.

The steel sample was pickled in a dilute solution of hydrochloric acid HCl 1:1 for 10 min in order to remove any possible oxide layer and the oily film from the surface. After that, this sample was rinsed off with ethanol to remove the residual hydrochloric acid layer. In the next step, the sample was immersed in an ultrasonic bath filled with alcohol for 5 min and then dried in air.

**Figure 3:**
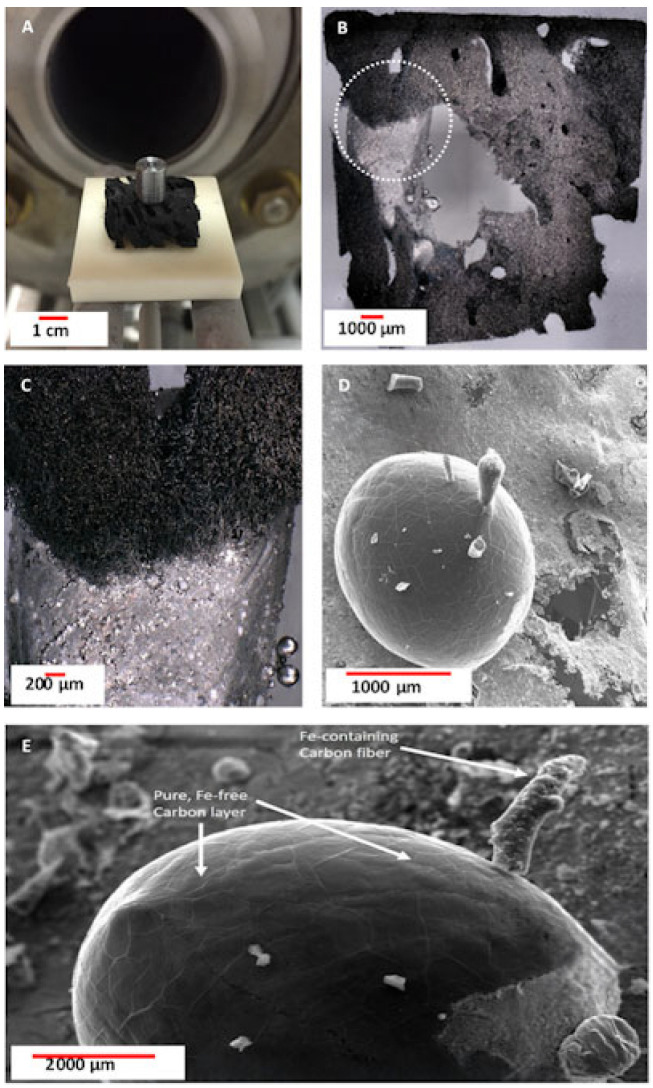
(**A**) Photograph of the experimental setup taken prior to the “steel on carbon” melting test shows that the cylindrical steel sample exhibited no visible surface deformation on the carbonized spongin scaffold. (**B**,**C**) The metallic phase that remains after steel melting can be observed within the carbonized spongin scaffold. (**D**,**E**) SEM images show the microarchitecture of the steel fragment built within the fibrous carbonized scaffold after melting.

A selected fragment (10 × 10 × 3 mm; m = 0.112 g) of already carbonized (1200 °C) spongin scaffold was placed at the center of an Al_2_O_3_-based ceramic plate. A cylindrical steel (1.4103, Table 2) sample (Ø = 3 mm, H = 5 mm; M = 0.564 g) was then positioned on top of this carbonized scaffold (Figure 3).

This construction was placed on two ceramic tubes so that it was located exactly above the end of the thermocouple. Subsequently, the table with the sample was pushed into the oven, which was closed with a clamping ring. The furnace was evacuated to a pressure of 1 mbar; after that, the flooding process was carried out with argon 5.0 at a flow rate of 2.0 L/min. Since the pressure in the furnace was about 1000 mbar, the argon’s volume flow was extended up to 7.0 l/min, and the valve of the argon outlet was opened to purge the furnace. The tube furnace was purged for 1 h with argon 5.0 at a flushing rate of 7.0 l/min. The data for the heating conditions of the oven (heating speed 2 °C/min, temperature limit 1450 °C, holding time 2 h, and cooling speed 10 °C/min) were entered in the controller. A temperature of 1450 °C was chosen as a representative value above the melting point of low-carbon steel, allowing full melting of the sample and its interaction with the carbonized spongin.

### 2.3. Characterization Techniques

#### 2.3.1. Digital Optical Microscopy

Selected samples were observed using an advanced imaging system, VHX-7000 digital optical microscope with zoom lenses VHX E20 (magnification up to 100×) and VHX E100 (magnification up to 500×) (Keyence, Osaka, Japan).

#### 2.3.2. Scanning Electron Microscopy (SEM) with Energy Dispersive X-Ray Analysis (EDX)/Elemental Mapping

SEM-EDX/elemental mapping analyses were carried out with a scanning electron microscope (Quanta 250 FEG; FEI Ltd., Brno, Czech Republic) equipped with an energy-dispersive X-ray spectrometer (Ametek/EDAX Team V.4.4.). The method was used to verify the elemental composition and surface morphology of the samples.

#### 2.3.3. Fourier Transform Infrared Spectroscopy

FT-IR spectra of the obtained materials were recorded using a Nicolet iS50 spectrometer (Thermo Fisher Scientific Co., Hillsboro, OR, USA) with a built-in attenuated total reflectance (ATR) accessory. The analyses were carried out in a wavelength range of 4000–400 cm^−1^.

#### 2.3.4. X-Ray Diffraction

X-ray analyses were performed using a powder diffractometer (SmartLab Rigaku, Tokyo, Japan) with a CuK alpha lamp in a 2-theta range of 3–120 (scan step 0.01, scan speed 4°/min).

#### 2.3.5. Magnetic Properties

The magnetic properties of the obtained materials were tested with a neodymium magnet of a pull force of 192 N (Mistral, Jaworzno, Poland).

Magnetic measurements were also carried out using a vibrating sample magnetometer (VSM, Poznań, Poland). All measurements were performed at room temperature within a magnetic field range of ±1 T. Data were collected for the pure steel and coated sponge-based samples.

#### 2.3.6. Transmission Electron Microscopy (TEM)

The samples were cut to a thickness of 80 nm with a glass knife and placed on copper grids covered with a carbon film. Measurements were conducted with a transmission electron microscope, Jeol JEM-ARM 200cF (JEOL (EUROPE) SA UPROSZCZONA, Warszawa, Poland), at an acceleration voltage of 100 kV. International Centre for Diffraction Data (ICDD) was used for phase identification, and the ICDD card no. 33-0664 for hematite.

### 2.4. Electrochemical Measurements

#### 2.4.1. Electrochemical Cell Configuration

The electrochemical performance was tested in 0.5 M Na_2_SO_4_ in a 3-electrode system, where the Pt sheet was utilized as the counter-electrode, and the chloro-silver electrode (Ag/AgCl/KCl_(sat.)_) was the reference electrode. All electrochemical measurements were performed using the Gamry Interface 5000 multichannel electrochemical system (Gamry Instruments, Warminster, PA, USA) at ambient temperature (22 ± 2 °C).

#### 2.4.2. Preparation of the Working Electrode

The carbonized spongin/stainless steel 316 L sample (CS + 316 L_STL) was ground in a mortar to standardize the current density in the electrochemical measurements. The resulting powder was then used to prepare the electrode mixture, which consisted of 80% *w*/*w* of the investigated material, 10% *w*/*w* conductive additive (carbon black), and 10% *w*/*w* polymer binder (polyvinylidene fluoride). N-methyl-2-pyrrolidone (NMP) was used as a solvent. The working electrode was prepared by carefully applying the mixture onto a glassy carbon electrode (GC; diameter = 3 mm) and then evaporating the solvent at 40 °C overnight.

The carbonized spongin/stainless steel 316 L sample (CS + 316 L_STL) was chosen for electrochemical testing due to its high hematite and residual nickel content, which may support the electrode’s catalytic properties. The investigated working electrode is later referred to as GC/CS + 316 L_STL.

#### 2.4.3. Electrochemical Measurements

Linear sweep voltammetry (LSV) was performed with a scan rate of 5 mV s^−1^, and corresponding Tafel plots were derived based on previous LSV measurements. All potentials measured were referenced to the reversible hydrogen electrode (RHE) using the equation: E*_(RHE)_* = E*_(Ag/AgCl/KCl(sat.))_* + 0.197 + 0.059 pH.

## 3. Results

Our initial studies on the use of spongin carbonized at 1200 °C indicate the potential for the formation of a 3D nanostructured composite based on Fe_2_O_3_. The high thermal stability of the spongin-derived framework enables the simultaneous formation of carbon material and nanoscale iron oxide structures, particularly following exposure to steel melting temperatures at 1450 °C. Notably, the distinctive fibrous architecture of spongin remains intact throughout both the pyrolysis and hydrothermal treatment processes conducted under these conditions.

**Figure 4:**
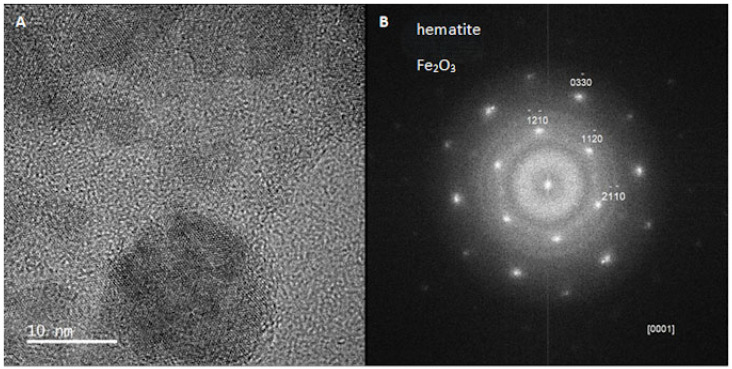
Transmission electron microscopic (TEM) image and electron diffraction pattern of nanoparticles obtained from carbonized spongin microfibers after the steel melting process. (**A**) TEM image of one hematite nanoparticle of ~20 nm in size, (**B**) electron diffraction pattern identified as hematite.

The surface of the remaining metal becomes coated with a carbon layer (Figure 3D,E) originating from carbon microfibres embedded deep within the metallic phase. Analytical studies of microfibres extracted from the metalized 3D structure reveal the emergence of magnetic properties. These properties can be attributed to a nanocrystalline metallic phase that is uniformly distributed both on the surface and throughout the microfibres. Notably, this phase remains firmly attached to the carbonized scaffold even after 30 min of ultrasonic treatment. High-resolution TEM and electron diffraction analyses confirmed the presence of this nanometallic phase, identifying it as hematite-type iron oxide (Figure 4).

### 3.1. Digital Optical Microscopy

**Figure 5:**
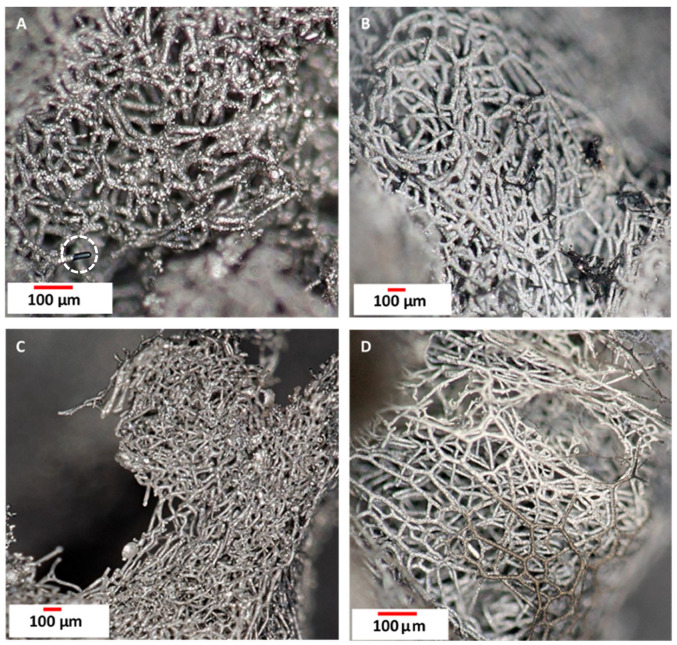
Digital optical microscope images of carbonized spongin (CS) samples after melting of selected steel samples at 1450 °C: (**A**) CS + construction steel EN S235JRG2 (AISI 1015); (**B**) CS + carbon steel C45; (**C**) CS + no. 172/1 low alloy cast iron; (**D**) CS + stainless steel 316 L powder.

**Figure 6:**
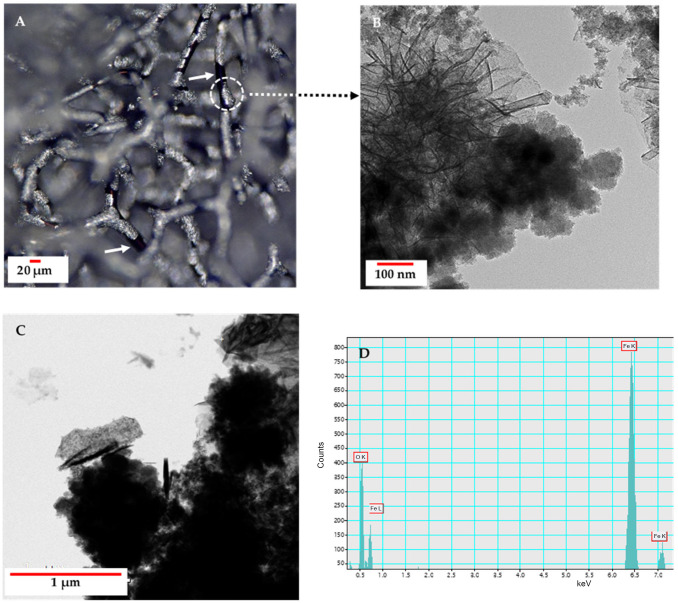
Digital microscopy image (**A**) of the carbonized spongin construct after carbon steel C45 melting on its surface shows fragments with a characteristic metallic luster. TEM images (**B**,**C**) represent structural peculiarities of crystals of iron oxide origin (EDX data, (**D**)).

Digital optical microscope images (Figure 5) represent carbonized spongin obtained from the original spongin scaffold treated with 3 M HCl (see Figure 1) after the melting of the selected steel samples (Figure 5A–D). The surface of these carbonized spongin fibers acquires a metallic luster as a result of the formation of a nearly homogeneous, microstructured, iron-containing layer. The surface of the carbonized spongin fibers remains black in the areas not covered with such a layer (Figure 5B,C). The thickness of the iron layer has been measured as 2.9 µm, taking into account the diameter of carbonized spongin fiber as 10 µm. Figure 6 represents the morphology of the iron oxide crystalline phase formed on the surface of carbonized spongin after steel melting.

### 3.2. Scanning Electron Microscopy (SEM) with Energy Dispersive X-Ray Analysis (EDX)/Elemental Mapping

**Figure 7:**
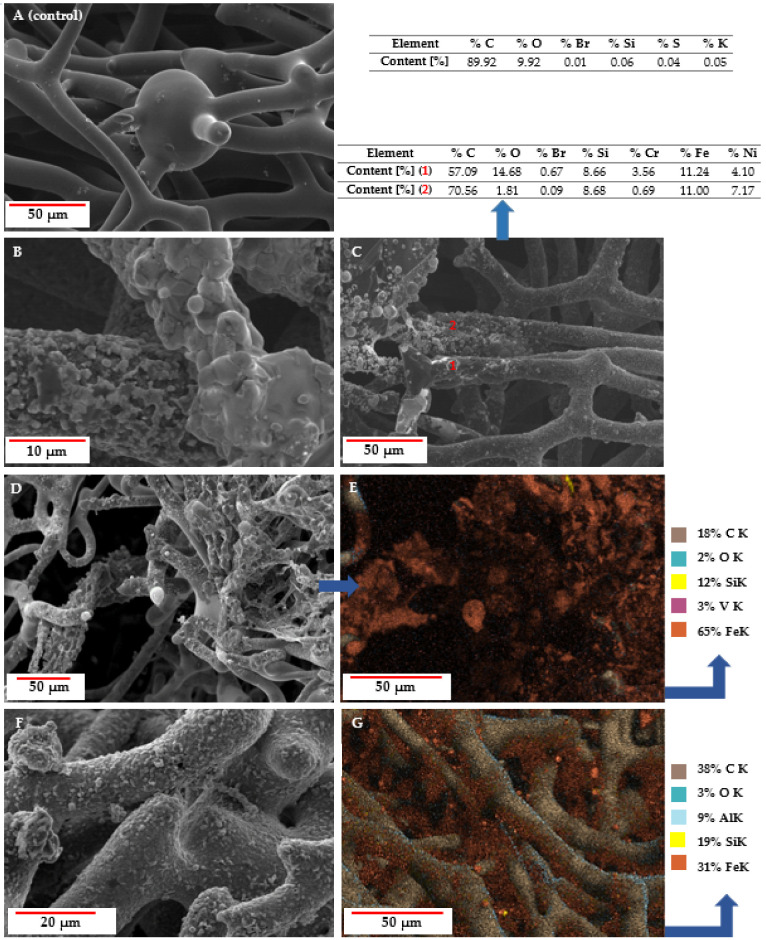
SEM images with EDS or elemental mapping analyses of carbonized spongin (CS) samples under study: (**A**) control sample CS (carbonized spongin scaffold at 1200 °C); (**B**) CS + construction steel EN S235JRG2 (AISI 1015) melted at 1450 °C; (**C**) CS + stainless steel 316 L powder melted at 1450 °C; (**D**) CS + carbon steel C45 melted at 1450 °C with (**E**) elemental mapping analysis; (**F**) CS + no. 172/1 low alloy cast iron melted at 1450 °C with (**G**) elemental mapping analysis.

SEM images (Figure 7) of the tested samples, EDX analysis, and elemental mapping indicate the presence of iron. This proves that the interaction between carbonized spongin and steel occurred during the melting process of steel on its surface. The detection of additional metals (i.e., Cr, Ni, and V) corresponds to the specific metal composition of the steel samples used in the process, while Si, Br, and S are known to be present in spongin [9].

**Figure 8:**
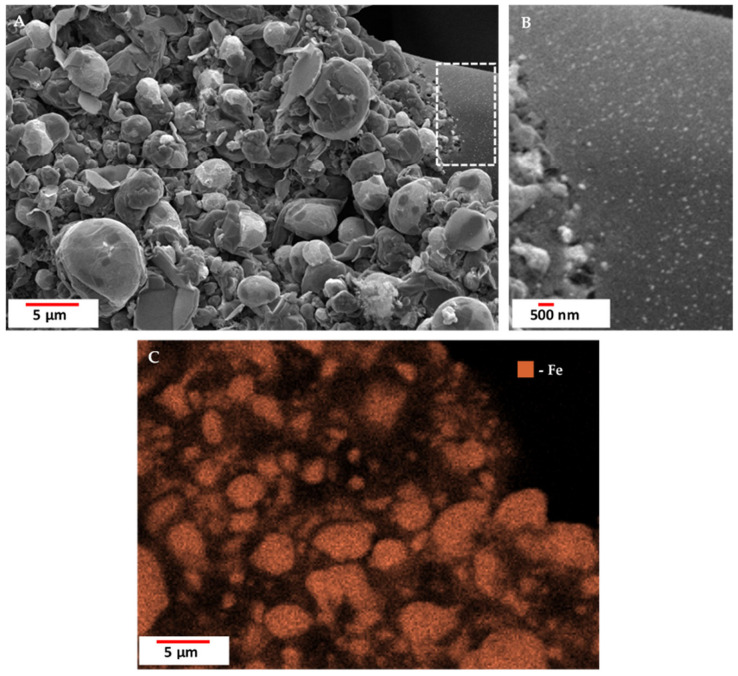
SEM images (**A**,**B**) with elemental mapping analyses (**C**) of spongin treated with 3 M HCl and next 40% HF carbonized at 1200 °C after the carbon steel C45 melting process at 1600 °C. The average diameter of iron nanoparticles in image B is 90.40 nm (see also Supplementary Materials, Figures S2–S5).

The results of the SEM analysis show that the surface of the carbonized spongin fibers after the melting of steel had a micro- and nano-grain structure (Figure 8). This structure consists of tightly bound iron-oxide particles ranging in diameter from 39 nm (see Supplementary Materials, Figure S2) to up to 5 µm. Although optical microscopy creates the illusion of near-homogeneous metallization of the fiber surface (Figure 5), scanning microscopy shows the heterogeneity of the metal layer in terms of granulation. Surface imperfections became visible after the steel melting process, with iron oxide grains merging with the fiber surface, creating both irregular and spherical structures (see also Supplementary Materials, Figures S2–S5). A similar phenomenon has been observed by Linderhof and co-workers [35] in their SEM study of ferritic phase transformation in stainless steel 1.4404 (CL20ES) and maraging steel 1.2709 (CL50WS) after selective laser melting.

**Figure 9:**
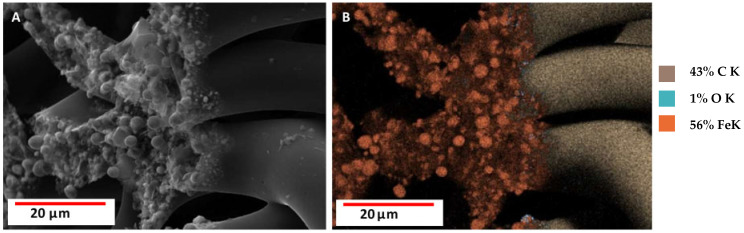
(**A**) SEM image and (**B**) elemental mapping analysis of the phenomenon of microfiber rupture followed by metallization of the inner surface of carbonized at 1200 °C spongin previously treated with 3 M HCl and next 40% HF after carbon steel C45 melting process at 1600 °C (see also Supplementary Materials, Figures S6 and S7).

Also, in all the studied samples, SEM revealed the formation of internally torn carbonized fibers densely dotted with iron oxide microparticles (see Figure 9, Figures S6 and S7 in Supplementary Materials). These images suggest that the inner surface of the carbonized spongin fibers also plays an active part in the formation of these micro and nanoparticles.

**Figure 10:**
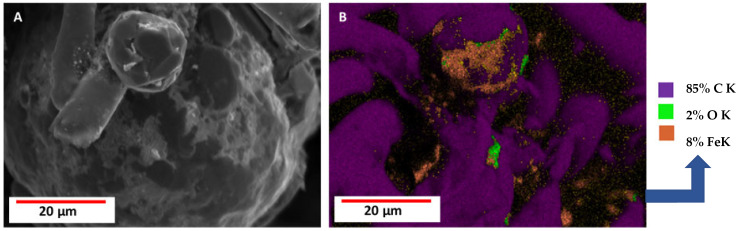
SEM image (**A**) with elemental mapping analysis (**B**) of carbonized spongin at 1200 °C previously treated with 3 M HCl and 40% HF after carbon steel C45 melting at 1600 °C. The iron-oxide-based microspheres are clearly visible as they are covered with a carbon layer (Supplementary Materials, Figure S8).

The formation of spherical structures was observed when the temperature of melted steel was increased to 1600 °C (Figure 10 and Figure S8 in Supplementary Materials). These structures featured a central core composed of an iron-rich phase, while their surfaces were coated with a nanolayer of carbon. A similar phenomenon was also observed at 1450 °C (Figures S6 and S7B–D in Supplementary Materials). The formation mechanism of these carbon films on the surface of iron oxide spherical structures remains unclear and requires further investigation.

### 3.3. Fourier Transform Infrared Spectroscopy

**Figure 11:**
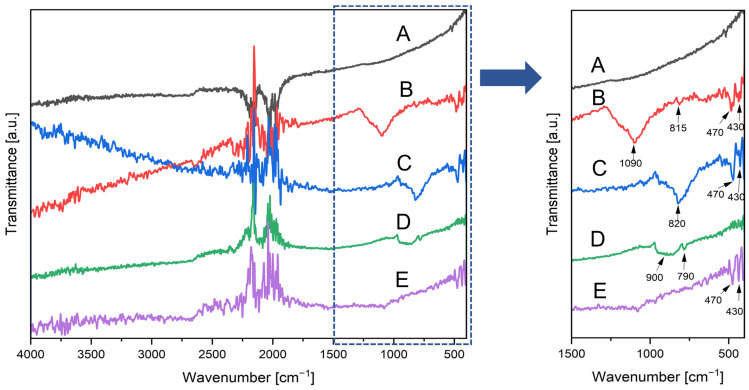
FT-IR spectra of control sample and samples after steel melting process (all spongin samples after 3 M HCl treatment before carbonization): (A) carbonized spongin (CS); (B) CS + stainless steel 316L, 1450 °C; (C) CS + construction steel EN S235JRG2 (AISI 1015), 1450 °C; (D) CS + carbon steel C45, 1450 °C; (E) CS + no. 172/1 low alloy cast iron, 1450 °C.

FT-IR analyses presented in Figure 11 were performed for carbonized spongin (control sample) and the carbonized spongin subjected to the steel melting process. The aim was to investigate the formation of iron-containing phases on the carbonized spongin matrix with different types of steel. Although IR analysis of carbonized spongin is generally limited due to the dominance of carbon-related signals and low IR activity, significant spectral changes were detected, suggesting interactions between the molten steel and the organic matrix.

FT-IR spectra of carbonized spongin samples with different types of molten steel (Figure 11) exhibit bands characteristic of iron oxide structures. In particular, FT-IR spectra of the samples obtained with 316L, AISI 1015, and no. 171.2 steel shows bands characteristic for Fe-O vibrations of the γ-Fe_2_O_3_ phase at 430 cm^−1^ and for Fe-O vibrations of the α-Fe_2_O_3_ phase at 470 cm^−1^ [36,37,38]. In the spectrum of carbonized spongin with C45 steel, the broad band observed at ~900 cm^−1^ is assigned to Fe-O stretching vibrations [39]. Additionally, the bands characteristic of Fe-O-H vibrations are observed around 800 cm^−1^ and can be shifted depending on the effects of the other components [39,40]. In the received spectra, this band is observed at 790 cm^−1^ for the sample with C45 steel, and at 815 cm^−1^ and 820 cm^−1^ for the sample with 316L and AISI 1015 steel, respectively, which suggests the presence of goethite in these samples. However, the bands at 815 cm^−1^ and 820 cm^−1^ in samples with 316L and AISI 1015 steel can also be related to SiO_2_ contamination [41,42]. A distinct band observed near 1090 cm^−1^ in spectra of carbonized spongin with 316L steel can be assigned as a C-O or Fe-O-C stretching vibration, indicating the formation of chemical bonds at the interface between iron oxide nanoparticles and the carbon matrix [43].

The FT-IR results are in line with the HRTEM and XRD findings on hematite presence in the obtained samples by confirming the presence of characteristic hematite (α-Fe_2_O_3_) band at 470 cm^−1^, except for the sample with C45 steel. However, the obtained results also suggest that the oxide component of the samples is multiphase—the bands characteristic for maghemite (γ-Fe_2_O_3_) and goethite (α-FeO(OH)) are also observed.

### 3.4. X-Ray Diffraction

**Figure 12:**
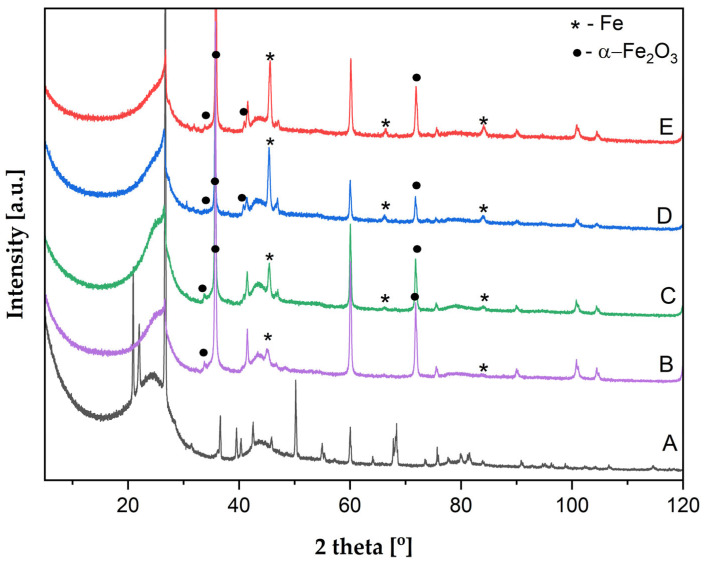
XRD patterns for the control sample (carbonized spongin) and samples after the steel melting process (samples after 3 M HCl treatment). (A) carbonized spongin (CS); (B) CS + stainless steel 316L, 1450 °C; (C) CS + no. 172/1 low alloy cast iron, 1450 °C; (D) CS + carbon steel C45, 1450 °C; (E) CS + construction steel EN S235JRG2 (AISI 1015), 1450 °C.

The X-ray diffraction pattern (Figure 12) of spongin carbonized at 1200 °C (control sample) is consistent with the results published previously (for details, see [15]). However, additional peaks originating from impurities are detected—for example, peaks at 22.2°, 31.5°, and 36.6° correspond to those of crystobalite SiO_2_ [44].

The diffraction patterns of carbonized spongin after the steel melting process show the characteristic peaks for Fe and (α-Fe_2_O_3_), marked with * and ●, respectively (Figure 12). Strong peaks observed for all metalized samples at 45.5° and weaker peaks at 66.3° and 84.9° are attributed to the metallic α-Fe phase—(110), (200), and (211) planes, respectively [45,46,47]. Peaks related to the Fe metallic phase are of the lowest intensity and plane (200) not detected for the sample prepared with 316 L stainless steel, while for the same sample, peaks related to hematite (α-Fe_2_O_3_) are stronger in comparison to other samples, which can suggest a higher contribution of hematite in this sample. The peaks characteristic of (104) (110), (113), and (101) planes of α-Fe_2_O_3_ are observed at 32.7°, 35.8°, 40.6°, and 71.8°, respectively [48,49,50,51,52].

### 3.5. Magnetic Properties

**Figure 13:**
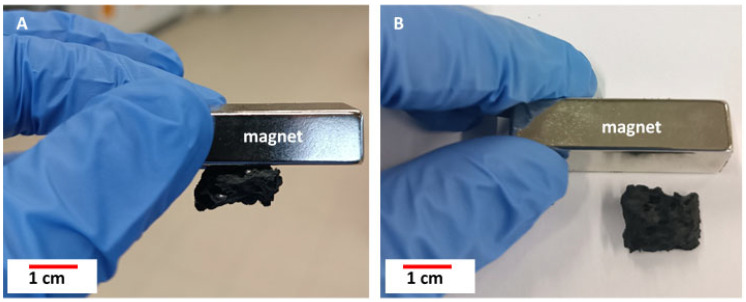
Samples attracted to the neodymium magnet: (**A**) CS + stainless steel 316 L, 1450 °C; (**B**) CS—control sample carbonized at 1200 °C.

All samples were attracted to the neodymium magnet, which also confirms the presence of magnetic iron (Figure 13, Video S1 in Supplementary Materials). According to the literature [53,54], Fe and nanoparticles of α-Fe_2_O_3_ are ferromagnetic compounds, highlighting the significant potential of the obtained composite materials for future applications involving magnetic functionality.

**Figure 14:**
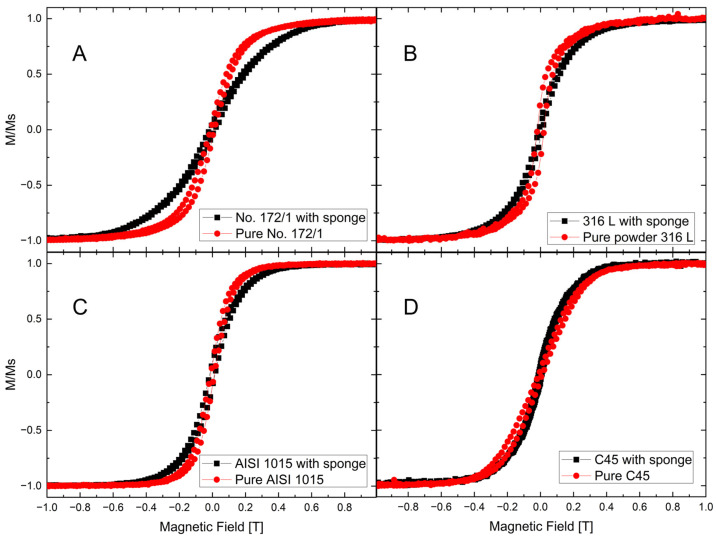
Normalized hysteresis loops for all investigated materials: reference steel (red) and coated carbon sponge (black): (**A**) no. 172/1 low alloy cast iron; (**B**) stainless steel 316 L powder; (**C**) construction steel EN S235JRG2 (AISI 1015); (**D**) carbon steel C45.

In VSM results, the hysteresis loops indicate that the reference materials retain their ferromagnetic properties when incorporated into the coated carbon sponge (Figure 14). Data were collected for the pure steel samples and the coated, sponge-based samples with dimensions of 1 cm^3^, i.e., about 103–104 times higher than the reference samples. It suggests that magnetic sponge samples exhibit ferromagnetic behavior due to an unoxidized metallic part of the coatings, since hematite is a canted antiferromagnet with a negligible magnetic moment [55]. The pure carbonized spongin did not exhibit magnetic properties as presented in the Supplementary Material (Figure S9).

Due to geometry and material quantity differences among the samples, the hysteresis loops were normalized to the saturation signal to better compare the saturation fields determined mostly by magnetic anisotropy, mainly the shape anisotropy (see characteristic “knees” in Figure 14). The hysteresis loops of both reference powder samples and the magnetic sponges do not differ much in their saturation fields of 0.2–0.3 T, which additionally confirms the “particle” nature of the ferromagnetic part of the coatings. A slight increase in the saturation field may also reflect a broader distribution of crystallite sizes in the coatings. To sum up, the magnetic behavior of the magnetic sponges may be attributed to the presence of fine metallic crystallites, as observed in the SEM analysis (Figure 7).

### 3.6. Catalytic Properties During the Hydrogen Evolution Reaction

**Figure 15:**
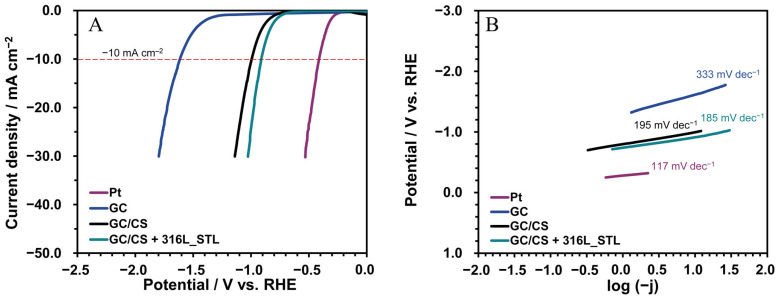
Results of the electrochemical characterization: (**A**) LSV curves; (**B**) the Tafel plots extracted from the corresponding LSV curves.

The electrode prepared as GC/CS + 316 L_STL was tested for its electrocatalytic performance in hydrogen evolution. The linear sweep voltammetry (LSV) polarization curves, recorded at a scan rate of 5 mV s^−1^ (Figure 15A), exhibit a typical shape for water reduction in neutral media (0.5 M Na_2_SO_4_). Notably, no additional peaks that would suggest any decomposition of the sample within the applied voltage range were observed. All investigated electrodes based on carbonized spongin (CS) had better electrocatalytic properties than the unmodified glassy carbon electrode (GC), for which the hydrogen evolution overpotential (η) at −10 mA cm^−2^ was the highest (1.6 V vs. RHE). The electrode made from untreated carbonized spongin (GC/CS) demonstrated good electrocatalytic properties, showing an overpotential (η) of 1.0 V vs. RHE at a current density of −10 mA cm^−2^. However, our proposed modification procedure, which involved forming a composite material with 316 L stainless steel, significantly enhanced its hydrogen reduction capabilities. The GC/CS + 316 L_STL electrode exhibited the lowest η value among all tested hybrid materials, with a measured η of 0.9 V vs. RHE at −10 mA cm^−2^. For comparison, the η for the Pt electrode was 0.4 V vs. RHE at −10 mA cm^−2^.

Figure 15B presents the Tafel plots corresponding to the LSV curves. The Tafel slope is an essential parameter for describing the reaction mechanisms of the catalysts [56]. At high overpotentials, the kinetically controlled hydrogen evolution reaction can be described by the Tafel equation: *η* = *a* + *b log j*. In this equation, *η* (V) represents the overpotential, *a* (V) is the cathodic intercept related to the exchange current density, *b* (V dec^−1^) refers to the Tafel slope, and *j* (A cm^−2^) denotes the catalytic current density [57]. The Tafel slope values calculated from the linear portion of the potential versus logarithmic current density curve were 117, 185, 195, and 333 mV dec^−1^ for Pt, GC/CS + 316 L_STL, GC/CS, and GC, respectively. Among the synthesized hybrid materials, GC/CS + 316 L_STL exhibited the lowest Tafel slope value, highlighting the positive effect of the transition metal residues observed in Figure 7C and described earlier in the literature (Table S1) [58,59,60,61,62,63,64,65,66].

## 4. Discussion

The phenomenon of metal evaporation at a minimum pressure of 1.8 mm Hg or lower vacuum [67] is used to deposit layers of a given metal on selected materials (i.e., metalized plastic film production) [68,69,70,71]. Analysis of the spongin-based composites obtained as a result of melting steel on carbonized spongin skeletons allows us to assume that, also, in an inert gas atmosphere (in this case, argon), the process of iron evaporation from liquid steel takes place after exceeding its melting temperature of 1450 °C. Iron ions react with carbon from the 3D carbonized spongin skeleton in the form of turbostratic graphite [15], creating a new composite material. We suggest that this steam also condenses on the surface of the carbonized skeleton, resulting in the deposition of nanostructured iron oxide (hematite) responsible for the created construct’s magnetic properties. Alternatively, iron ions react with the surface of the turbostratic graphite of sponge origin [10,15]. This causes the formation of nucleation points, which are of significant importance for the growth and development of iron oxide nanoparticles visible under SEM observations (Figure 7). It can be speculated here that also previously reported ferromagnetic properties of diverse turbostratic graphites [72] and HOPG [73,74,75], could be involved in the adsorption of iron ions on the surface of graphite-based spongin composite under study. However, such ferromagnetic properties should be investigated in future research.

There is limited understanding of the physics behind the iron/graphite interfacial behaviors. Yajun et al. [76] demonstrated a strong correlation between graphite morpholo-gy, liquid flow dynamics in the lower region of the blast furnace, and the interfacial energy between iron and graphite. In their research, molecular dynamics (MD) simulations were used to investigate the preferential positioning of anti-spheroidizing elements, such as oxygen atoms, in melt-cast iron and iron and its influence on iron/graphite interfacial energy. The results revealed that oxygen atoms tend to adsorb more readily onto the prism planes of graphite rather than the basal planes. Furthermore, the presence of these adsorbed oxygen atoms was found to significantly alter the wettability of molten iron on the prism plane, resulting in a notable reduction in interfacial energy as the number of adsorbed oxygen atoms increased [76].

Several scientific groups have previously investigated the interaction of graphite with iron ions and oxides. For example, special effects have been reported for the ion implantation method, which offers the insertion of foreign species, including iron ions, into such host material as graphite. Koon and co-workers reported that for ions as heavy as iron implanted at 100 keV, an amorphous layer on the graphite surface results in fluences as low as 1 × 10^14^ atoms/cm^2^ [77]. Commonly, chemical reactions occur via either reduction or functionalization, where iron nanoparticles are covalently bound to the graphite surface [75]. According to the classical view [78], the interaction of graphite with iron in a liquid state or vapor leads to the formation of graphite intercalatim compounds (GICs) in which metal atoms donate their electrons to the carbon layers. In this case, the reverse donor-acceptor complexes are formed with negatively charged carbon layers and metallic cations between them [78]. More than 90% of the iron in these complexes has been found as π-complexes. It has been reported that iron atoms in the GIC with Fe form a bilayered structure within the interlayer space [78].

It was observed that iron oxide selectively deposits on defects and step edges of highly oriented pyrolytic graphite (HOPG) when using iron chloride (FeCl_2_ · 4H_2_O) as a precursor, leading to the formation of iron oxide nanoparticles without requiring any additional chemical processing. These nanoparticles, supported on the HOPG surface, were subsequently annealed in air at temperatures ranging from 100 °C to 500 °C for 2 h [75].

The formation of α-Fe_2_O_3_ and Fe_3_O_4_-pillared graphites has been reported by Morishige and Hamada as a result of pillaring graphite oxide with trinuclear iron acetate complex ions and calcining it in air and in vacuo, respectively. It was shown that mesopores of Fe_3_O_4_-pillared graphite constitute a hydrophobic nanospace [79]. Diverse iron oxide functionalized graphite oxide pre-catalysts have been synthesized via a rapid and facile γ-Fe_2_O_3_ nanoparticle incorporation into the functional carbon nanosheets [80]. Studies involving iron-implanted graphite have shown that embedding iron atoms between graphite layers is significantly more energetically favorable than their adsorption ad atom onto the surface [81]. Additionally, iron ad atoms exhibit high mobility both across the graphite surface and within its bulk structure [81]. The method for developing iron oxide nanoparticles coated expanded graphite as adsorbents by the hydrothermal decoration approach for phosphorus removal from aqueous solution has also been represented [82].

Pyrolysis of diverse, rich carbon precursors of biological origin in the presence of an iron catalyst in an inert atmosphere remains one of the methods for producing nanostructured graphitic carbons (for details, see [83]). Recently, the creation of the turbostratic carbon/Fe_2_O_3_ composite synthesized by catalytic graphitization of sucrose has been reported [84].

Owen and co-workers developed a series of iron oxide-functionalized graphene and graphite oxide pre-catalysts through a rapid and straightforward method involving the incorporation of γ-Fe_2_O_3_ nanoparticles into functionalized carbon nanosheets [80]. The graphene oxide framework was initially prepared by exfoliating graphite, after which well-characterized γ-Fe_2_O_3_ nanoparticles, averaging 50 nm in size, were integrated into the nanostructure. This process resulted in an innovative pre-catalyst scaffold suitable for further activation using H_2_ and CO_2_/H_2_ reactions aimed at efficient CO_2_ conversion [80].

A series of iron oxide functionalized graphene and graphite oxide pre-catalysts were synthesized via a rapid and facile γ-Fe_2_O_3_ nanoparticle incorporation into the functional carbon nanosheets by Owen and co-workers [80]. The graphene oxide synthetic scaffold was first synthesized by the exfoliation of graphite. Subsequently, iron nanoparticles (γ-Fe_2_O_3_) with well-understood morphologies and dimensions (i.e., averaging 50 nm) were introduced into the nanostructure, thus yielding a novel pre-catalyst scaffold, ready for a subsequent pre-activation by H_2_ and CO_2_/H_2_ reactions for a rapid CO_2_ conversion [80].

Agaogularii et al. [85] synthesized magnetic iron nanoparticles enclosed in graphite from a solid precursor containing hematite and graphite using a combined ball milling and low-pressure chemical vapor deposition method. Such nanoparticles have a high potential for use in medicine due to their high magnetic and catalytic abilities. They also show high chemical resistance, as they are not degraded in strong acids, such as HCl and HF.

Fayazi et al. [72] proved that ferromagnetic ordering occurs in turbostratic graphite samples with vacancies and boundary defects. Moreover, heat treatment of this material causes an increase in the degree of turbostratic arrangement of graphite and, consequently, an increase in ferromagnetism in the sample. Such graphite-based materials can be used in devices based on magnets and data recording. Thambiliyagodage et al. [84] and Gomez-Martin et al. [86] carried out catalytic graphitization of naturally occurring sucrose by incorporating different amounts of iron to obtain sucrose–Fe_2_O_3_ nanocomposites. Studies have shown that turbostratic graphite is formed in the presence of iron, and the degree of graphitization increases with increasing iron loading. The ordered turbostratic carbon/Fe_2_O_3_ exhibits dye adsorption capacity and photocatalytic activity upon exposure to sunlight. Gomez-Martinez et al. [86] proved that the graphitization temperature depends on the local structure of amorphous carbon and the size and oxidation state of the catalyst. After the deposition of the iron precursor, its decomposition occurs as a result of reduction from Fe_2_O_3_ to Fe_3_O_4_ and then to FeO at a temperature below 700 °C. At 700 °C, Fe is formed, and as a result of CO_2_ oxidation, Fe_3_C is formed, which initiates graphitization. The greatest structural development occurs in the range of 1000–1600 °C; above this value, no greater ordering of the structure is observed.

Andris et al. [87] studied the interaction of graphite with metal surfaces. It was confirmed that the chemical reactions of graphite particles with metal alloys used in the thermal annealing process decrease adhesive strength. The presence of graphite affects the corrosion process and the formation of oxide layers on alloys (iron oxide in the case of the 800H alloy). Stoneham [88] showed that iron particles undergo complex movements on the graphite surface, i.e., uniform Brownian movements of catalyst particles. The shape of the catalyst changes during the movement, as does the temperature of the catalyst, even when the substrate temperature is kept constant. Luo et al. [89] showed the synthesis of graphite-coated Fe_3_O_4_-FeC_x_ nanoparticles to catalyze the conversion of CO_2_ to light olefins. Defects in the graphite coating of the core-shell catalyst have a beneficial effect on CO_2_ conversion.

Nizhenko and Floka [90] investigated the interfacial interactions between graphite and various liquid Fe-based alloys, including Fe–Cu, Fe–Sn, Fe–Ge, Fe–Ga, and Fe–Al. In their experiments, a cylindrical iron sample was positioned on a flat graphite substrate and heated until melting. At each test temperature, the molten alloy remained in contact with the graphite surface for 15 min, during which the contact angle (θ_1_) was periodically measured [90].

The interaction of iron vapor with fullerite and graphite substrates causes changes in the structure of iron–pearlite, cementite, and graphite deposits formed. The wettability of carbon materials by molten metals depends on the type of metal. The work of Nikonova et al. [91] has shown that the shape of the iron drop stays on the substrate surface after melting the metal on the fullerite and graphite substrate, while nickel began to penetrate the graphite substrate and completely penetrated the fullerite substrate. Copper, on the other hand, did not react with carbon structures at all.

Composites obtained by melting steel on carbonized spongin matrices will also find a number of applications due to their unique properties, but this still requires a lot of research. Ultimately, the composites obtained in this way will find application in the extreme conditions of modern industry on a large scale. It is worth noting that the obtained composite materials have magnetic properties, which means that, in addition to typical industrial applications, they can also be used in electronics, environmental protection, and even aviation. However, one of the most promising yet uncharted territories for applying such materials is the generation of green hydrogen from electrochemical water splitting for use as a sustainable energy source [58].

Briefly, water electrolysis involves two main processes: the oxygen evolution reaction (OER) and the hydrogen evolution reaction (HER) [92]. The HER takes place at the cathode of the electrolyzer and can produce high-purity hydrogen. When the electrochemical cell is powered by renewable energy sources, this hydrogen is viewed as an environmentally friendly energy carrier. The key challenge in HER is the development of cost-effective electrocatalysts [93]. These catalysts should lower the overpotential of the cathodic reactions, reducing the high energy consumption of the electrochemical water-splitting process. Thus, it would be a more attractive alternative to methods based on fossil fuels, such as steam methane reforming. Currently, platinum and ruthenium dioxide are widely recognized as the leading catalysts for the HER due to their exceptional electrocatalytic activity [94]. However, the limited availability and high cost of these noble metals significantly restrict their commercial applications. Therefore, well-abundant transition metals such as cobalt, nickel, molybdenum, and iron can serve as suitable alternatives to noble metals due to their catalytic activity and relatively low cost [58,92,95].

The layered structure of graphite and its derivatives enhances electron mobility, which is advantageous for catalytic reactions such as the HER. As a result, much of the research on HER catalysts focuses on using graphite as a convenient foundation for developing complex composite materials. For example, layered electrodes made from metallic NiCo-nitrides/NiCo_2_O_4_ and graphite fibers have shown catalytic activities that are comparable to those of platinum-based electrodes [96]. Simultaneously, iron oxides have attracted significant interest for their application in water electrolysis. This is primarily due to their high stability, environmental friendliness, and favorable electrochemical activity [58]. However, iron oxides are often reinforced by other transition metals to enhance the efficiency of HER [59].

Matrices used for overall water-splitting catalysts are often carbon-based heterogeneous materials, including zero-dimensional carbon black, one-dimensional carbon nanotubes, and two-dimensional graphene [97]. However, the technological implementation of these catalysts, which require a high surface area, can be challenging. In this study, we present a cost-effective two-step method for preparing a carbonized spongin/hematite/nickel compound catalyst that preserves the unique 3D structure of the *H. communis* spongin scaffold. Since the metal alloy used in this synthesis can be chosen freely, this material offers an attractive platform for further optimization. Even more efficient HER catalysts could be achieved by future modifications, such as doping with other transition metals [98] or integrating additional catalytic phases [99]. Ultimately, this research contributes to the development of sustainable and scalable materials for green hydrogen production.

## 5. Conclusions

Extreme biomimetics is an incredibly fascinating area of research that not only mimics natural processes under extreme conditions but also shows ways to use existing equipment for common high-temperature processes for biological materials to create new and sometimes unexpected composite materials. As a renewable marine biopolymer with a unique 3D microarchitecture, spongin already plays a key role in creating new composite materials within the framework of modern scaffolding strategies of bioinspired materials science. In this work, magnetic sponge-like composites based on a metalized scaffold of carbonized 3D skeletons of cultivated marine sponge *H. communis* were created for the first time. Previously treated with HCl, the carbonized spongin scaffolds were used as matrices for melting diverse types of steel on their surface. Their microtopography and composition analysis revealed that the created materials consist of graphite cores coated with an iron oxide-containing layer on a uniform submicron scale. However, further investigations reveal the expense for the next improvement of the coating process, aiming to avoid undiscovered elements observed during electron microscopic investigations. The composition analysis revealed that the developed composites contain the hematite phase after melting each type of steel used in the experiments. This shows the repeatable phenomenon of hematite creation while melting steel on carbonized spongin. Such magnetic graphite–hematite composites are potential candidates for environmental purification, biomedicine, and electrochemistry applications.

## Figures and Tables

**Table 1:** Composition of steels used in the study.

Type of Steel	% C	% Si	% Mn	% Fe	% Mo	% Cr	% Ni	% O	% V
AISI 1015	3.54	0.11	0.61	95.74	-	-	-	-	-
C45	27.57	0.16	-	65.15	-	0.14	-	6.98	-
316 L powder	3.83	1.12	-	64.94	0.88	19.07	10.16	-	-
No. 172/1	51.52	1.40	0.64	34.73	0.15	-	-	11.54	0.02

**Table 2:** Chemical composition of the steel sample 1.4301 in (mass %) and N * in (mass ppm).

Element	% Fe	% Si	% Cr	% Mn	% Ni	% C	% S	% P	% Ti	N *
Mass [%]	70.638	0.460	18.300	1.380	9.160	0.020	0.010	0.030	0.002	-
Mass [ppm]	-	-	-	-	-	-	-	-	-	443

## Data Availability

The raw data supporting the conclusions of this article will be made available by the authors on request.

## Supplementary Materials

The following supporting information can be downloaded at https://www.mdpi.com/article/10.3390/biomimetics10060350/s1: Figure S1: SEM images of steel samples: (A) construction steel EN S235JRG2 (AISI 1015); (B) carbon steel C45; (C) stainless steel 316 L; (D) no. 172/1 low alloy cast iron; Figure S2: SEM images (A, C) with elemental mapping analyses (B, D) of spongin treated with 3 M HCl and 40% HF carbonized at 1200 °C after carbon steel C45 melting process at 1600 °C; Figure S3: SEM image of spongin treated with 3 M HCl and 40% HF carbonized at 1200 °C after carbon steel C45 melting process at 1600 °C (enlargement of Figure S3C); Figure S4: SEM images (A, B) of spongin treated with 3 M HCl and 40% HF carbonized at 1200 °C after carbon steel C45 melting process at 1600 °C; Figure S5: SEM images (A, C, E) with elemental mapping analyses (B, D, F) of spongin treated with 3 M HCl and 40% HF carbonized at 1200 °C after carbon steel C45 melting process at 1600 °C; Figure S6: SEM images of the phenomenon of microfiber rupture followed by metallization of the inner surface of spongin treated with 3 M HCl and 40% HF carbonized at 1200 °C after steel melting process at 1450 °C: (A, B, C, D) construction steel EN S235JRG2 (AISI 1015); Figure S7: SEM images of the phenomenon of microfiber rupture followed by metallization of the inner surface of spongin treated with 3 M HCl and 40% HF carbonized at 1200 °C after steel melting process at 1450 °C and at 1600 °C: (A) carbon steel C45 at 1600 °C, (B, C, D) stainless steel 316L at 1450 °C. Figure S8: SEM images (A, C) with elemental mapping analyses (B, D) of spongin treated with 3 M HCl and 40% HF carbonized at 1200 °C after carbon steel C45 melting process at 1600 °C; Figure S9. VSM results for pure carbonized spongin. Video S1: Samples attracted to the neodymium magnet: (A) CS after 3 M HCl + stainless steel 316 L 1450 °C; (B) CS after 3 M HCl—control sample carbonized at 1200 °C. Table S1. Key electrochemical properties of different HER catalysts based on iron oxides.
