# Supplementary material for: Development of Magnetic Sponges Using Steel Melting on 3D Carbonized Spongin Scaffolds Under Extreme Biomimetics Conditions
Source: Biomimetics (Basel). 2025 May 28;10(6):350. doi: 10.3390/biomimetics10060350 (PMC12190833; doi:10.3390/biomimetics10060350)
Supplement: Supplementary file 1 [file biomimetics-10-00350-s001.zip › biomimetics-3609202-Supplementary materials_Bartosz Leśniewski.pdf]

biomimetics

Supplementary information for article

Development of magnetic sponges using steel melting on 3D carbonized spongin scaffolds under extreme biomimetics conditions

Bartosz Leśniewski [1,2,*], Martin Kopani [3], Anna Szczurek [1], Michał Matczak [4], Janusz Dubowik [4], Martyna Kotula [1,2], Anita Kubiak [1,2], Dmitry Tsurkan [5], Eliza Romańczuk-Ruszuk [6], Marek Nowicki [1,7], Krzysztof Nowacki [8], Iaroslav Petrenko [9] and Hermann Ehrlich [1,10,*]

1. Center for Advanced Technologies, Adam Mickiewicz University, Uniwersytetu Poznanskiego 10, 61-614 Poznan, Poland; anna.szczurek@amu.edu.pl (A.Sz.); markot6@amu.edu.pl (M.K.); anikub@amu.edu.pl (A.K.)
2. Faculty of Chemistry, Adam Mickiewicz University, Uniwersytetu Poznanskiego 8, 61-614 Poznan, Poland
3. Institute of Medical Physics and Biophysics, Faculty of Medicine, Comenius University, Sasinkova 2, 81272 Bratislava, Slovakia; martin.kopani@fmed.uniba.sk (M.K.)
4. Institute of Molecular Physics, Polish Academy of Sciences, Mariana Smoluchowskiego 17, 60–179 Poznań, Poland; michmat88@ifmpan.poznan.pl (M.M.); janusz.dubowik@ifmpan.poznan.pl (J.D.)
5. Institute of Nanoscale and Biobased Materials, Faculty of Materials Science and Material Technology, Technische Universität Bergakademie Freiberg, 09599 Freiberg, Germany; tsurkandd@gmail.com (D.T.)
6. Faculty of Mechanical Engineering, Institute of Biomedical Engineering, Bialystok University of Technology, Wiejska Str. 45C, 15-351 Bialystok, Poland; e.romanczuk@pb.edu.pl (E.R.-R.)
7. Institute of Physics, Faculty of Materials Engineering and Technical Physics, Poznan University of Technology, Piotrowo 3, 60-965 Poznan, Poland; marek.nowicki@amu.edu.pl (M.N.)
8. Faculty of Chemical Technology, Institute of Chemistry and Technical Electrochemistry, Poznan University of Technology, Berdychowo 4, 60-965 Poznan, Poland; krzysztof.nowacki@put.poznan.pl (K.N.)
9. Vice-Rectorate for Research, International Affairs and Transfer; Freiberg University of Mining and Technology; Akademiestrasse 6, D-09599 Freiberg, Germany; iaroslav.petrenko@zuv.tu-freiberg.de (I.P.)
10. Faculty of Chemical Technology, Institute of Chemical Technology and Engineering, Poznan University of Technology, Berdychowo 4, 60-965 Poznan, Poland

* Correspondence: barles5@amu.edu.pl (B.L.); herehr@amu.edu.pl (H.E.)

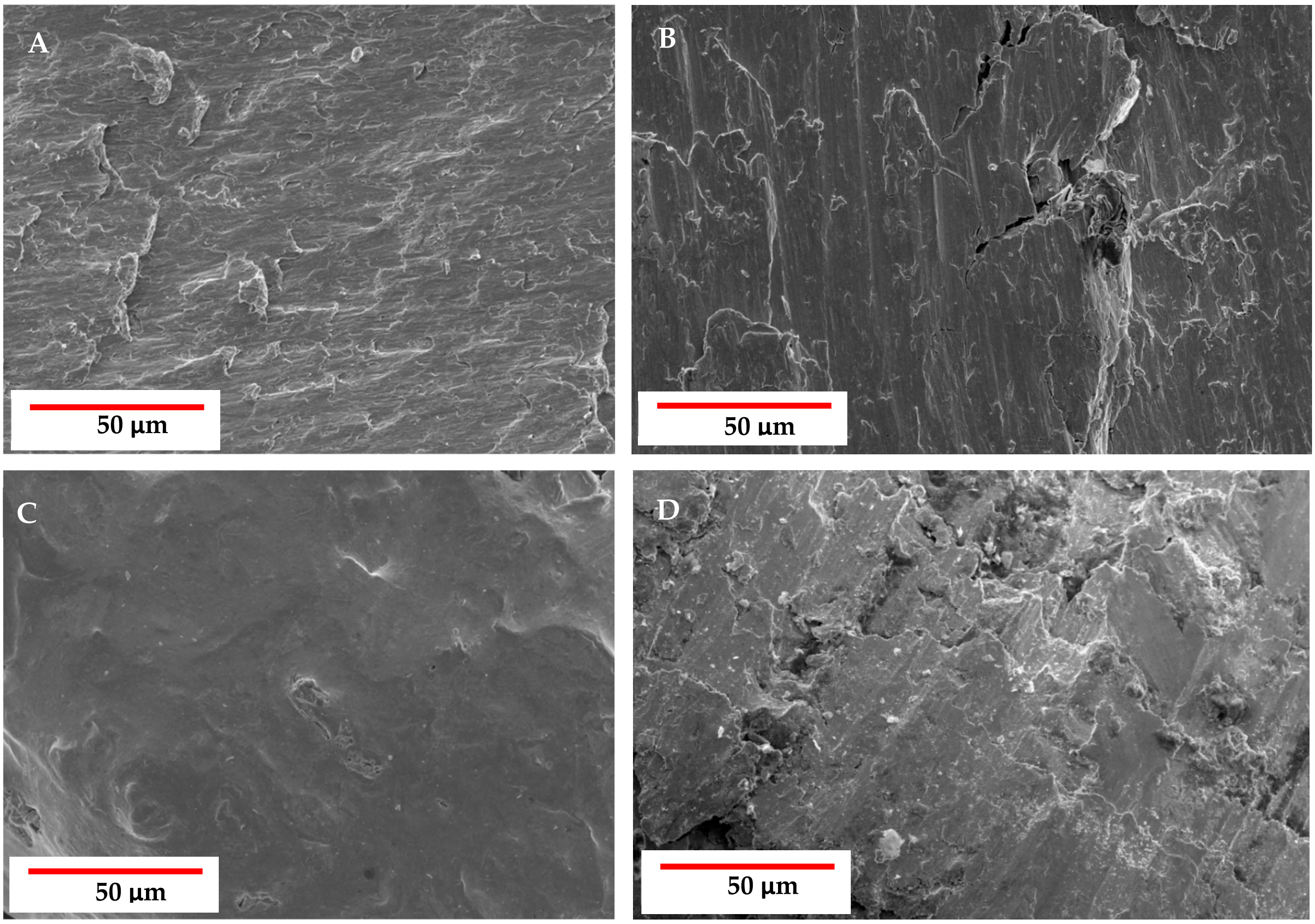

Figure S1. SEM images of steel samples: (**A**) construction steel EN S235JRG2 (AISI 1015); (**B**) carbon steel C45; (**C**) stainless steel 316 L; (**D**) no. 172/1 low alloy cast iron.

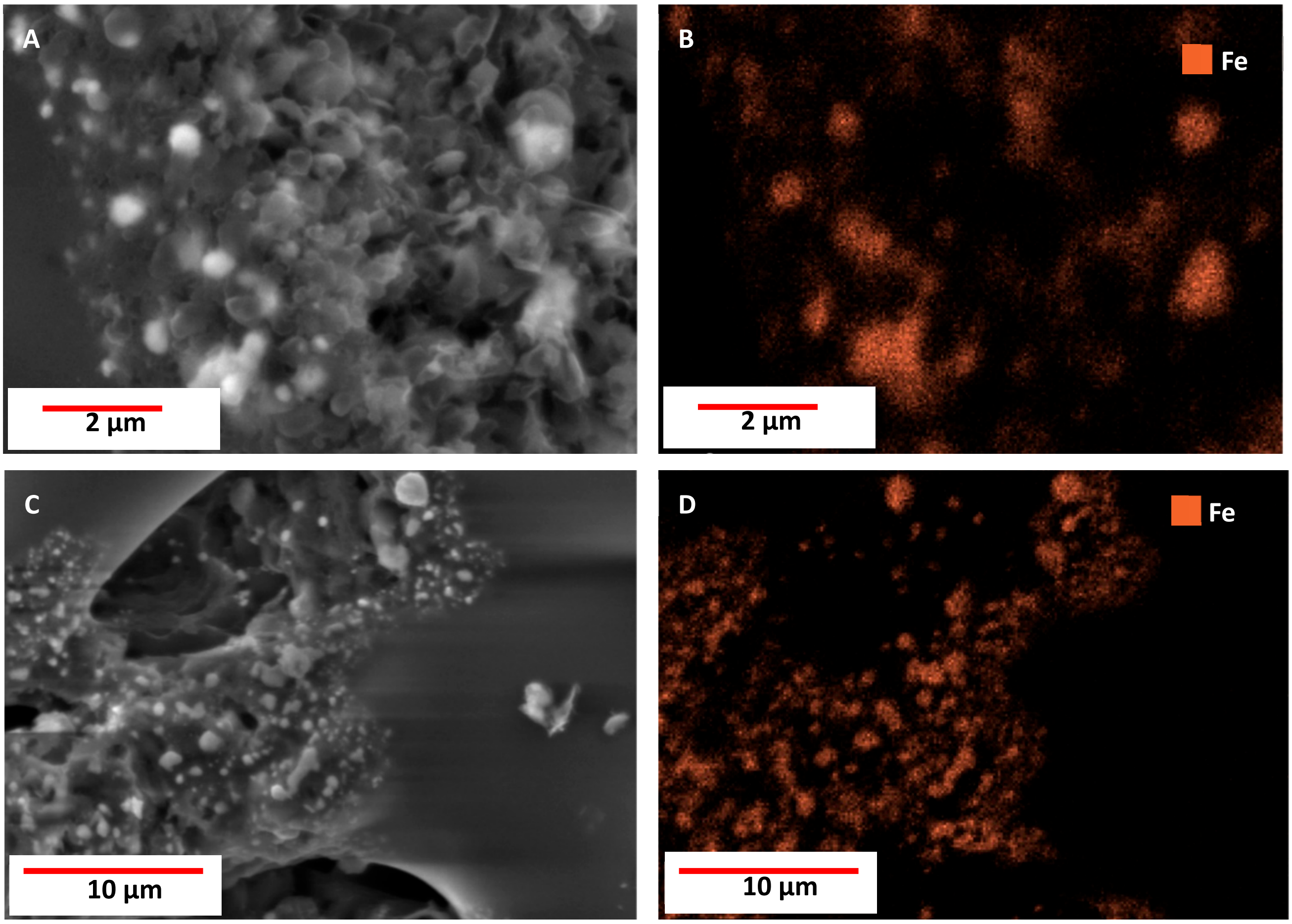

Figure S2. SEM images (**A, C**) with elemental mapping analyses (**B, D**) of spongin treated with 3 M HCl and 40 % HF carbonized at 1200 °C after carbon steel C45 melting process at 1600 °C.

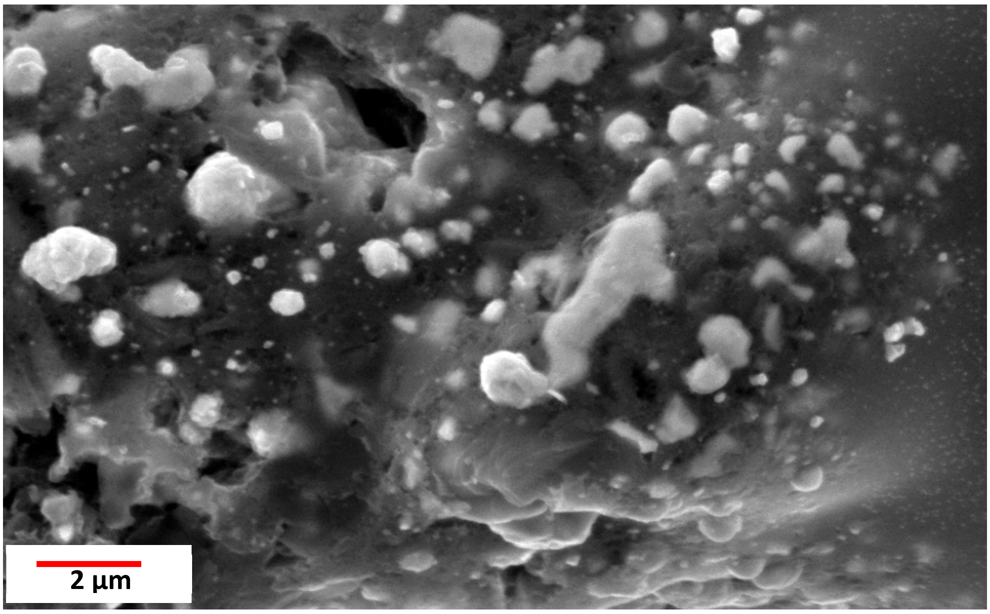

Figure S3. SEM image of spongin treated with 3 M HCl and 40 % HF carbonized at 1200 °C after carbon steel C45 melting process at 1600 °C (enlargement of Figure S3C) – average size of nanoparticles is 152.4 nm.

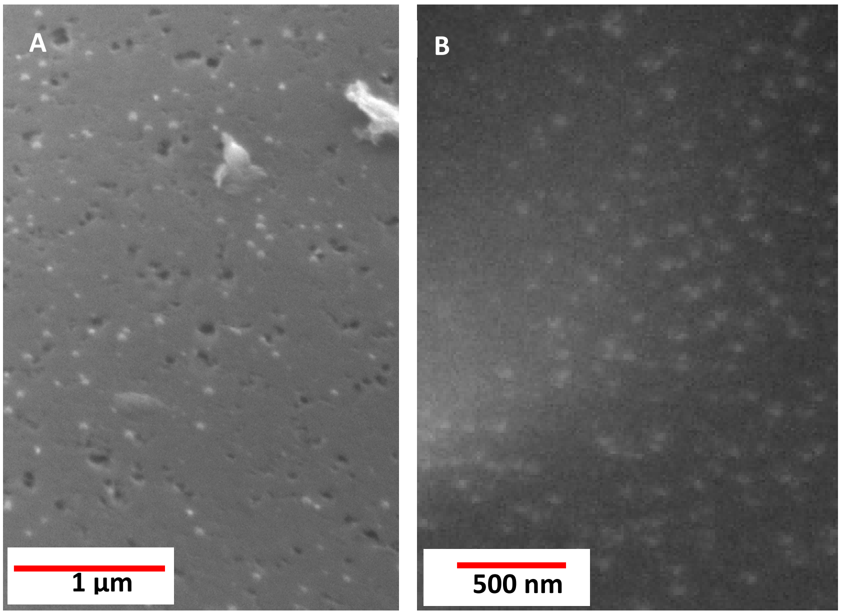

Figure S4. SEM images (**A, B**) of spongin treated with 3 M HCl and 40 % HF carbonized at 1200 °C after carbon steel C45 melting process at 1600 °C - average size of nanoparticles is 39.43 nm (image A) and 38.91 nm (image B).

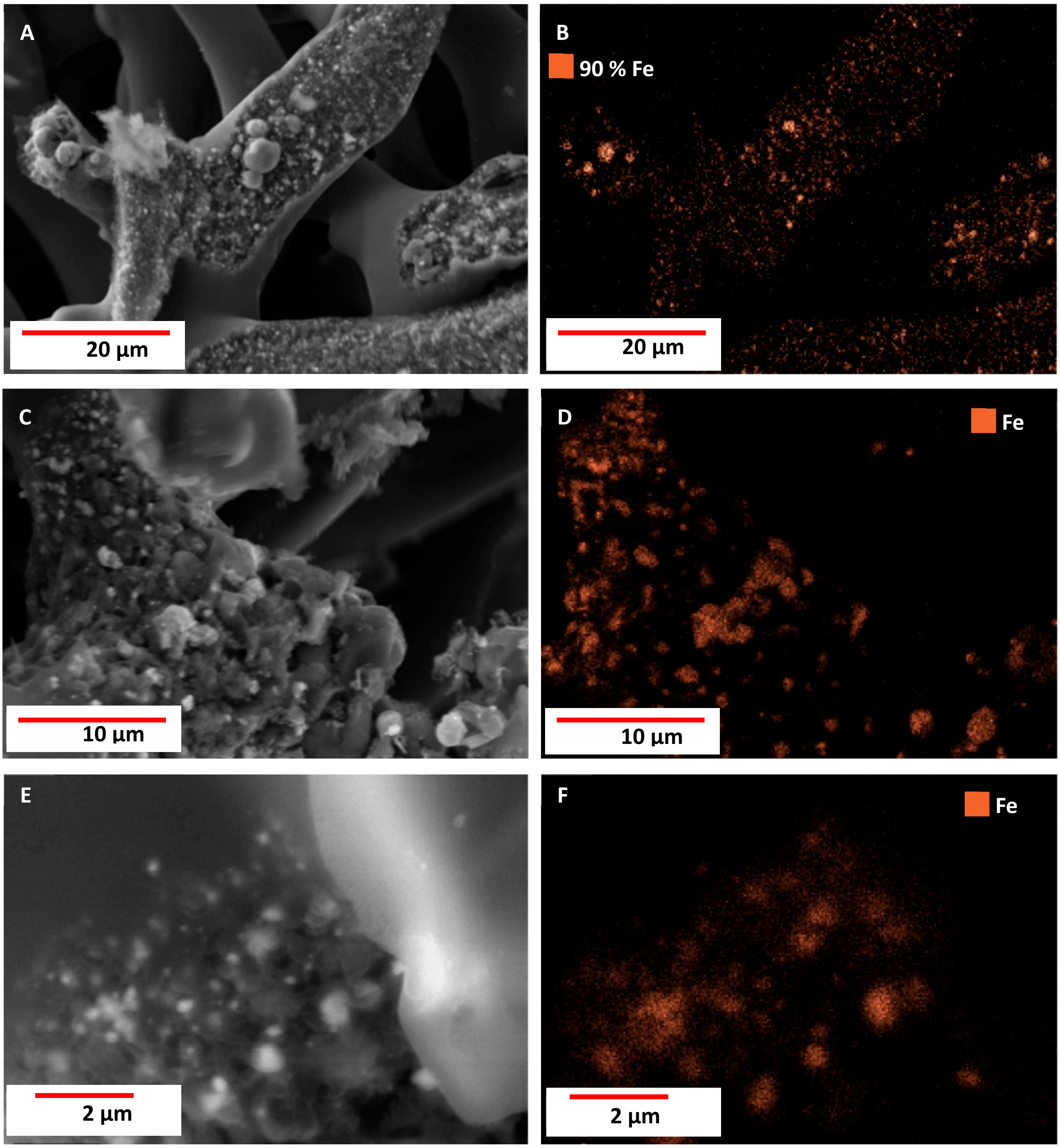

Figure S5. SEM images (**A, C, E**) with elemental mapping analyses (**B, D, F**) of spongin treated with 3 M HCl and 40 % HF carbonized at 1200 °C after carbon steel C45 melting process at 1600 °C.

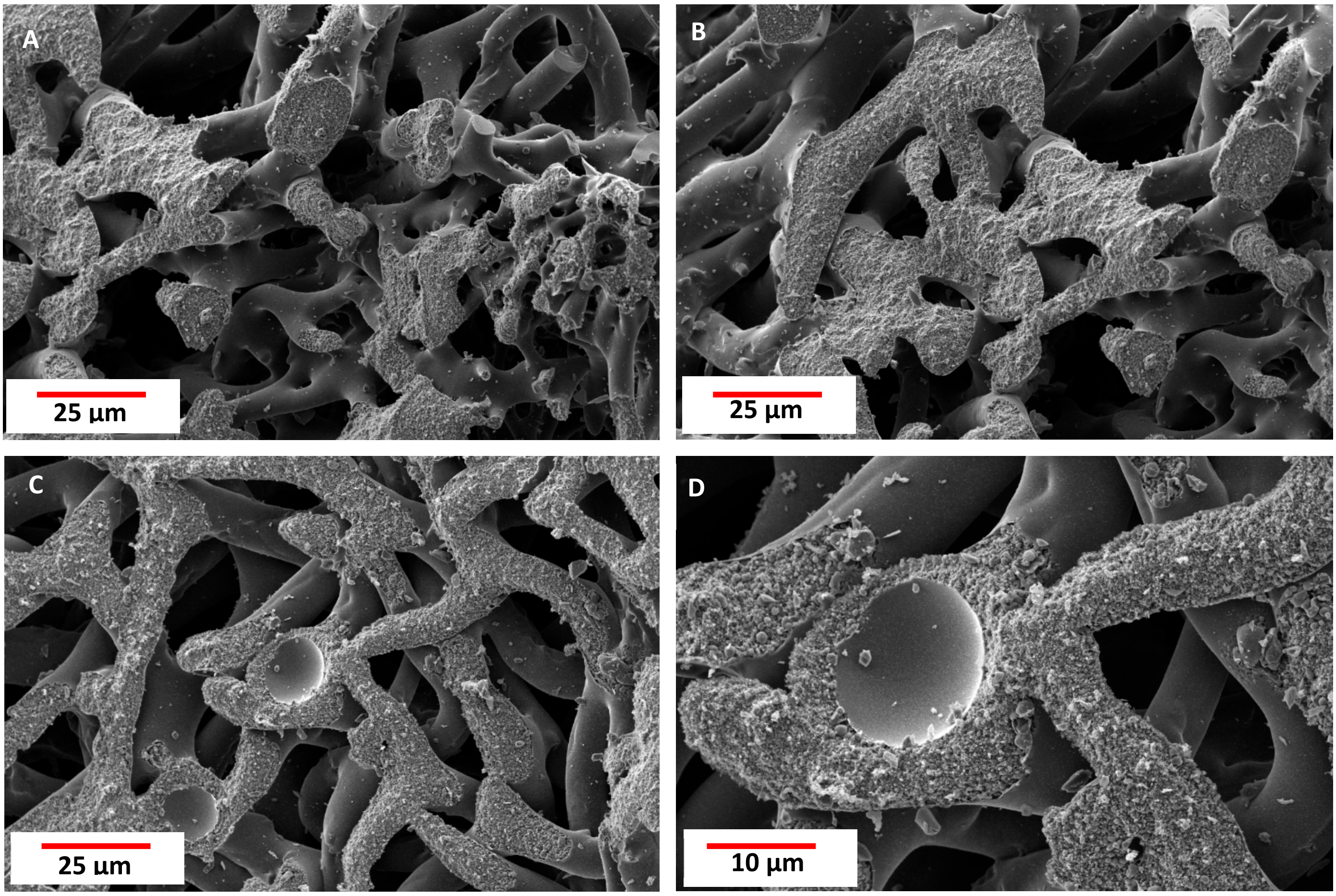

Figure S6. SEM images of the phenomenon of microfiber rupture followed by metallization of the inner surface of spongin treated with 3 M HCl and 40 % HF carbonized at 1200 °C after steel melting process at 1450 °C: (**A, B, C, D**) construction steel EN S235JRG2 (AISI 1015).

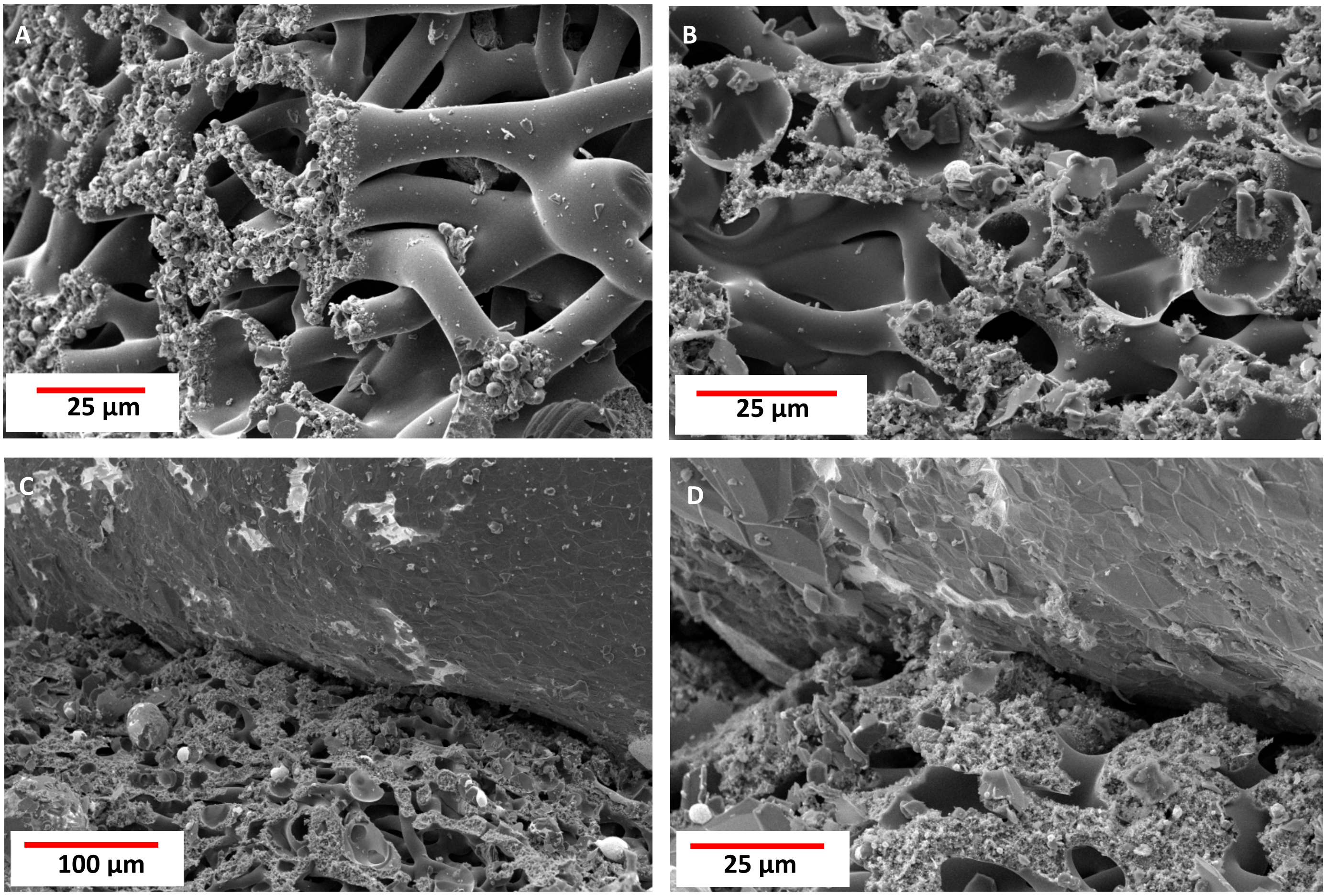

Figure S7. SEM images of the phenomenon of microfiber rupture followed by metallization of the inner surface of spongin treated with 3 M HCl and 40 % HF carbonized at 1200 °C after steel melting process at 1450 °C and at 1600 °C: (**A**) carbon steel C45 at 1600 °C, (**B, C, D**) stainless steel 316L at 1450 °C.

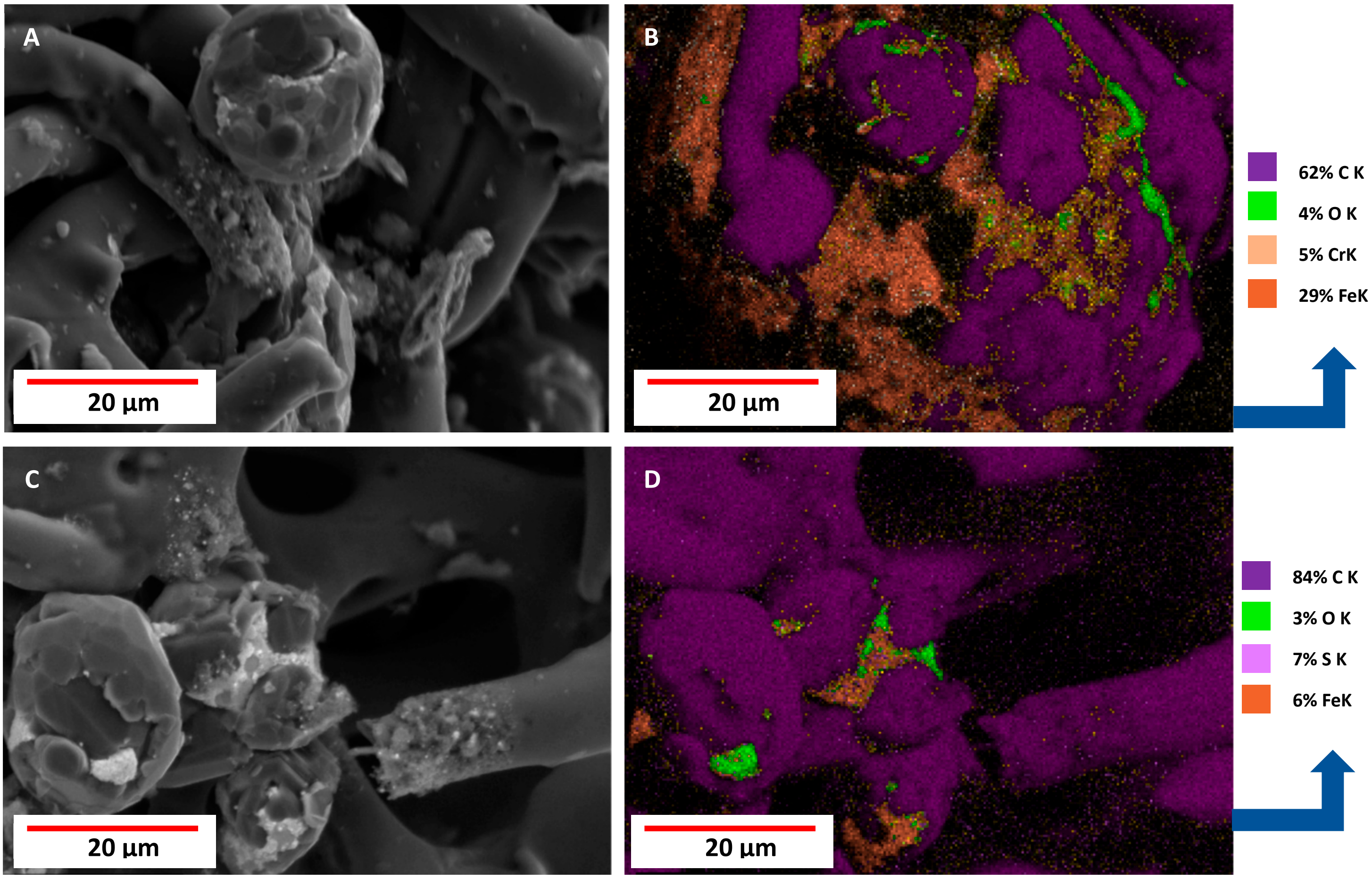

Figure S8. SEM images (**A, C**) with elemental mapping analyses (**B, D**) of spongin treated with 3 M HCl and 40 % HF carbonized at 1200 °C after carbon steel C45 melting process at 1600 °C.

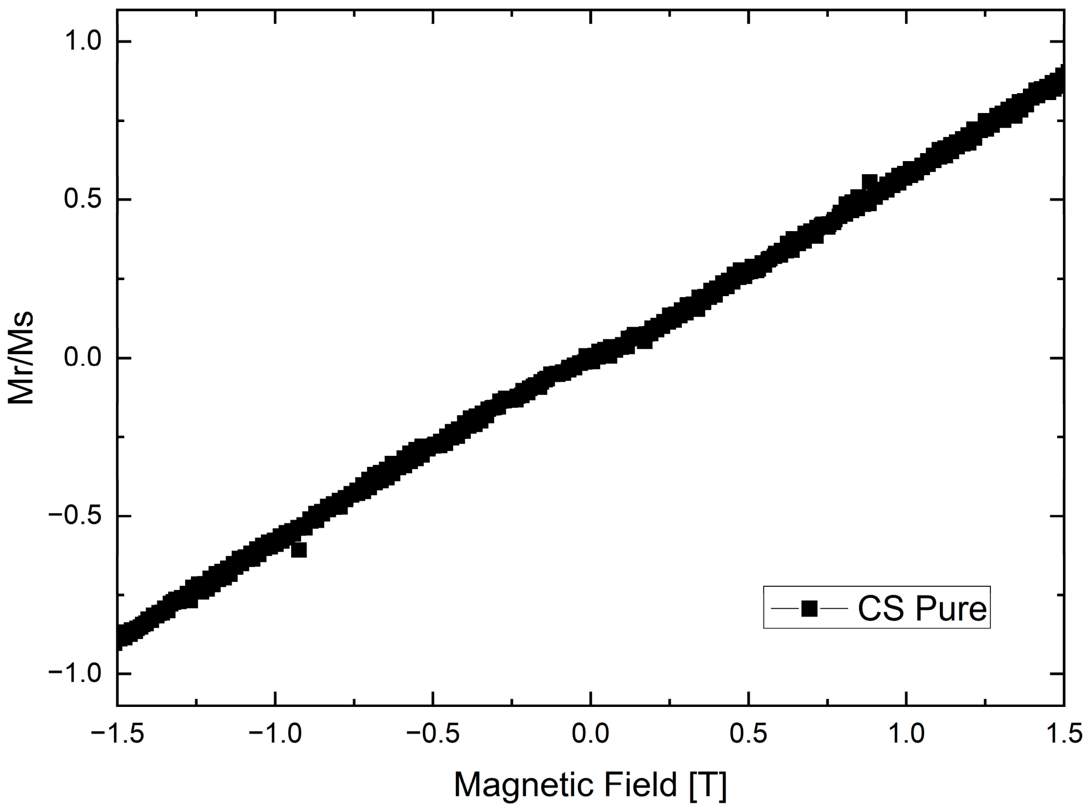

Figure S9. VSM results for pure carbonized spongin.

Table S1. Key electrochemical properties of different HER catalysts based on iron oxides.

Catalyst	HER overpotential (η; at –10 mA cm^{-2}) / V	Tafel slope / mV dec^{-1}	Electrolyte	Ref.
Carbonized spongin + α-Fe_2O_3 + Ni	0.9 vs. RHE	185	0.5 M Na_2SO_4	This work
Graphitic carbon nitride + Ce + Fe_2O_3	0.100 vs. RHE	81.5	1 M KOH	[58]
Nickel foam + CoMo + Fe_2O_3	0.071 vs. RHE	85	NaOH	[59]
Nickel foam + carbon + Fe_2O_3	0.211 vs. RHE	178	KOH	[60]
Iron foam + $NiFe_2O_4$/Fe_2O_3	0.041 vs. RHE	40.5	1 M KOH	[61]
MOF (MIL-53(Al)) + polypyrrole/Fe_2O_3	0.161 vs. RHE	57.9	Artificial seawater	[62]
Bio-based porous carbon + α-Fe_2O_3	0.250 vs. RHE	101.1	1 M KOH	[63]
MWCNTs + $CoFe_2O_4$	0.47 vs. RHE (from graph)	172	0.1 M K_2HPO_4/ KH_2PO_4	[64]
MWCNTs + α-Fe_2O_3	0.61 vs. RHE (from graph)	214	0.1 M K_2HPO_4/ KH_2PO_4	[64]
Nickel foam + Fe_2O_3 + P-doped $CoMoO_4$	0.068 vs. RHE (at –100 mA cm^{-2})	22.8	1 M KOH	[65]
Iron foam + Fe_2O_3	0.4 vs. RHE (from graph)	218.6	1 M KOH	[66]

References:

58. Anjana, R.; Hanamantrao, D.P.; Nasrin Banu, G.; Raja, V.; Isaac, R.S.R.; John, J.S.; Vediappan, K.; Jose, S.P.; Neppolian, B.; Sajan, D. Hydrothermal synthesis of graphitic carbon nitride/Ce doped Fe_2O_3 heterostructures for supercapattery device and hydrogen evolution reaction. *J. Energy Storage* **2025**, *116*, 116021.

59. Tong, H.; Zheng, X.; Qi, M.; Li, D.; Zhu, J.; Jiang, D. Synergistically coupled CoMo/Fe_2O_3 electrocatalyst for highly efficient and stable overall water splitting. *J. Colloid Interface Sci.* **2024**, *676*, 837–846.

60. Shetti, R.S.; Sreenivasulu, M.; Maiyalagan, T.; Alibrahim, K.A.; Alodhayb, A.N.; Shetti, N.P. Enhanced electrocatalytic performance of in situ pyrolized iron oxide-embedded carbon composite for sustainable hydrogen production. *Diam. Relat. Mater.* **2025**, *155*, 112305.

61. Li, L.; Wang, K.; Lei, T. Petal-like $NiFe_2O_4$/Fe_2O_3 heterostructure nanoarrays as bifunctional electrocatalyst for highly efficient alkaline overall water splitting. *Inorg. Chem. Commun.* **2025**, *173*, 113801.

62. Saadh, M.J.; Jasim, D.J.; Alejandro, L.; Saraswat, S.K.; Arévalo, C.G.F.; Brito, N.A.E.; Zainul, R.; Hasan, M.A.; Islam, S. Harnessing the potential of MOF/Fe_2O_3 nanocomposite within polypyrrole matrix for enhanced

hydrogen evolution. *Electrochim. Acta* **2024**, *507*, 145157.

63. Li, X.; Yang, Z.; Zhao, X.; Gao, M.; Liu, Y. Bio-derived mesoporous carbon confinement synthesis of ultra-small α-Fe_2O_3 nanoparticles as electrocatalysts for overall water splitting. *J. Electroanal. Chem.* **2024**, *973*, 118644.

64. Ibarra, J.; Aguirre, M.J.; del Río, R.; Henriquez, R.; Faccio, R.; Dalchiele, E.A.; Arce, R.; Ramírez, G. α-Fe_2O_3/, Co_3O_4/, and $CoFe_2O_4$/MWCNTs/Ionic Liquid Nanocomposites as High-Performance Electrocatalysts for the Electrocatalytic Hydrogen Evolution Reaction in a Neutral Medium. *Int. J. Mol. Sci.* **2024**, *25*, 7043.

65. Wang, B.; Chen, X.; He, Y.; Liu, Q.; Zhang, X.; Luo, Z.; Kennedy, J. V.; Li, J.; Qian, D.; Liu, J.; et al. Fe_2O_3/P-doped $CoMoO_4$ electrocatalyst delivers efficient overall water splitting in alkaline media. *Appl. Catal. B Environ.* **2024**, *346*, 123741.

66. Li, L.; He, C.; Lei, W.; Gao, P.; Lei, T. In situ synthesis of Fe_2O_3/Fe_3O_4 nanoarray hybrid as highly effective electrocatalysts for alkaline hydrogen evolution. *J. Alloys Compd.* **2024**, *978*, 173501.
