# Supplementary figures and images for: Development of Magnetic Sponges Using Steel Melting on 3D Carbonized Spongin Scaffolds Under Extreme Biomimetics Conditions
Source: Biomimetics (Basel). 2025 May 28;10(6):350. doi: 10.3390/biomimetics10060350 (PMC12190833; doi:10.3390/biomimetics10060350)

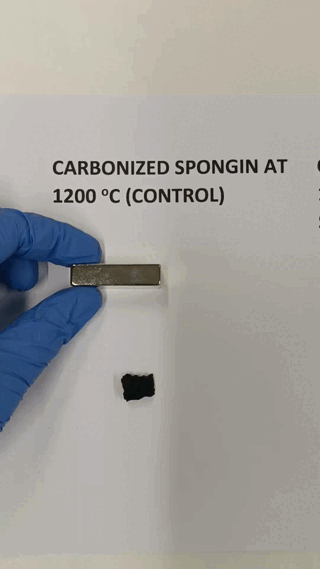

Supplement: Supplementary file 1 [file biomimetics-10-00350-s001.zip › Video S1.gif]
